# Microbiota and functional analyses of nitrogen-fixing bacteria in root-knot nematode parasitism of plants

**DOI:** 10.1186/s40168-023-01484-3

**Published:** 2023-03-10

**Authors:** Ye Li, Shaonan Lei, Zhiqiang Cheng, Lingyue Jin, Ting Zhang, Lian-Ming Liang, Linjie Cheng, Qinyi Zhang, Xiaohong Xu, Canhua Lan, Chaojun Lu, Minghe Mo, Ke-Qin Zhang, Jianping Xu, Baoyu Tian

**Affiliations:** 1 Provincial University Key Laboratory of Cellular Stress Response and Metabolic Regulation and College of Life Sciences, Fujian Normal University, Fuzhou, Fujian 350108 China; 2 State Key Laboratory for Conservation and Utilization of Bio-Resources in Yunnan and The Key Laboratory for Southwest Microbial Diversity of the Ministry of Education, Yunnan University, Kunming, Yunnan 650091 China; 3 Library, Fujian Normal University, Fuzhou, 350108 Fujian China; 4 Department of Biology, McMaster University, Hamilton, ON L8S 4K1 Canada

**Keywords:** *Meloidogyne*, Root-knot nematode, Parasitism, Root-associated microbiota, Nitrogen-fixing bacteria, Nitrogen fixation, Nematode-microbe-plant interaction

## Abstract

**Background:**

Root-knot nematodes (RKN) are among the most important root-damaging plant-parasitic nematodes, causing severe crop losses worldwide. The plant rhizosphere and root endosphere contain rich and diverse bacterial communities. However, little is known about how RKN and root bacteria interact to impact parasitism and plant health. Determining the keystone microbial taxa and their functional contributions to plant health and RKN development is important for understanding RKN parasitism and developing efficient biological control strategies in agriculture.

**Results:**

The analyses of rhizosphere and root endosphere microbiota of plants with and without RKN showed that host species, developmental stage, ecological niche, and nematode parasitism, as well as most of their interactions, contributed significantly to variations in root-associated microbiota. Compared with healthy tomato plants at different developmental stages, significant enrichments of bacteria belonging to Rhizobiales, Betaproteobacteriales, and Rhodobacterales were observed in the endophytic microbiota of nematode-parasitized root samples. Functional pathways related to bacterial pathogenesis and biological nitrogen fixation were significantly enriched in nematode-parasitized plants. In addition, we observed significant enrichments of the *nifH* gene and NifH protein, the key gene/enzyme involved in biological nitrogen fixation, within nematode-parasitized roots, consistent with a potential functional contribution of nitrogen-fixing bacteria to nematode parasitism. Data from a further assay showed that soil nitrogen amendment could reduce both endophytic nitrogen-fixing bacteria and RKN prevalence and galling in tomato plants.

**Conclusions:**

Results demonstrated that (1) community variation and assembly of root endophytic microbiota were significantly affected by RKN parasitism; (2) a taxonomic and functional association was found for endophytic nitrogen-fixing bacteria and nematode parasitism; and (3) the change of nitrogen-fixing bacterial communities through the addition of nitrogen fertilizers could affect the occurrence of RKN. Our results provide new insights into interactions among endophytic microbiota, RKN, and plants, contributing to the potential development of novel management strategies against RKN.

Video Abstract

**Supplementary Information:**

The online version contains supplementary material available at 10.1186/s40168-023-01484-3.

## Background

Plants harbor taxonomically and functionally diverse assemblies of microbial communities in their rhizosphere, rhizoplane, phylloplane, and endosphere [1]. These microorganisms and their plant hosts interact, co-adapt, and establish intimate relationships ranging from beneficial to commensal or harmful interactions [2, 3]. Together, plants and their associated microbiomes form a “holobiont,” which can confer plant hosts fitness advantages, such as growth promotion, resistance to biotic and abiotic stresses, and protection from pathogens and pests [2, 4]. However, growing evidence from recent studies suggests that pathogens or parasites are often accompanied with changes in community structure and function of plant-associated bacteria [5–9]. These bacteria may also negatively impact host plant health by producing toxins, suppressing plant innate immunity, and/or enhancing the survival and reproduction of plant pathogens and/or pests [5, 9–13]. Hence, the microbiota can influence not only the overall plant health but also key pathogenesis and parasitism processes.

Root-knot nematodes (RKN, *Meloidogyne* spp.) are among the most economically important plant-parasitic pests that can cause severe damages and yield losses in a wide variety of crops [14, 15]. After hatching from eggs in soil, the second-stage juveniles (J2) have a brief mobile stage before penetrating the root tips of plants. The nematodes then complete most of their life cycle inside the roots of the host plant [14, 15]. Inside these tissues, the nematodes move to the vascular cylinder and induce the formation of specialized feeding sites called “giant cells”, which act as the only nutrient source for the invading nematodes and provide all required resources for the growth, development, and reproduction of these nematodes [16–20]. The formation of giant cells and the proliferation of tissues surrounding the nematode feeding site lead to the disruption of the root system, which hinders its ability to uptake water and minerals and deliver them to other plant tissues and organs [16, 17, 21]. As obligate parasites, RKN depend completely on the supply of nutrients from the plants [16, 19]. The root system disruption in late stage of parasitism also raises the question about the ability of RKN to acquire enough nutrients from hosts, to fulfill the increasing demand for their development and late-stage reproduction [16, 17, 21].

Sharing a similar niche, RKN maintain an intimate relationship with the plant-associated microbiota throughout their life cycle [22–24]. Rich and diverse bacterial communities have been found associated with RKN, including all developmental stages, from eggs to mobile and infective larvae, till the completion of the specialized structure, the gall [24–27]. Several investigations on the rhizosphere microbiota of nematode-parasitized plants showed that nematode parasitism often enriched plant-beneficial bacteria, such as certain species of *Pseudomonas*, *Bacillus*, Actinomycetes, etc [22, 26, 28–30]. Furthermore, in nematodes associated with roots and within galls caused by *M. incognita*, enrichments of lignocellulose-degrading and nitrogen-fixing bacteria have also been observed [22, 24]. However, their potential roles have not been elucidated. In nature, eukaryote hosts establish symbiotic or mutualistic relationships with many associated microorganisms that are essential for the survival and reproduction of their hosts [22, 31–33]. The contributions by microorganisms are especially important for herbivorous insects, which mostly acquire nitrogen-poor nutrients from plant phloem sap. For example, aphids re-use the waste nitrogen compounds of bacterial symbionts to complement their nitrogen-poor diet from plant sap [11, 12]. As obligate RKN parasites feed on root giant cells as the sole source of nutrients, the nitrogen-poor nutrient from plant phloem sap, coupled with the observation of nitrogen-fixing bacteria within the root knots, led us to hypothesize that nitrogen-fixing bacteria may contribute to RKN parasitism by supplementing some nitrogenous nutrients for the nematodes [22, 24, 27, 30].

In this study, we investigated the changes in community composition of plant-associated microbiota during RKN parasitism. We identified keystone members of microbial communities that were taxonomically and functionally associated with RNK parasitism. Subsequently, functional contributions of the identified bacteria associated with RKN parasitism development were elucidated and validated.

## Results

### Effects of nematode parasitism on root-associated microbiota

**Fig. 1:**
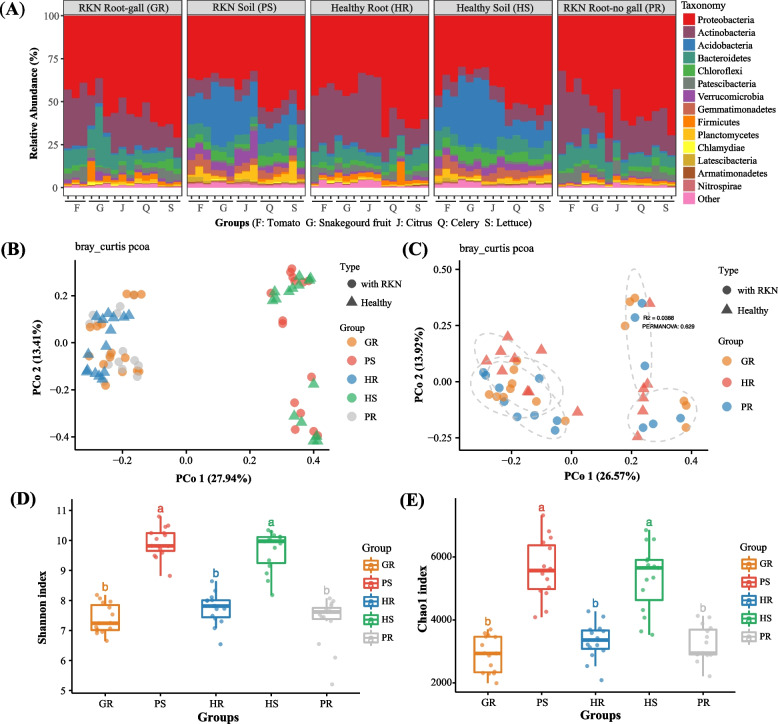
Effect of nematode parasitism on root-associated bacteria in five plant species.** A** Composition and relative abundance of major bacterial taxa in five plant species at the phylum level. **B** Unconstrained PCoA (PCo1 and PCo2) with Bray–Curtis distance showing the root-associated microbiota of the healthy and RKN-parasitized samples. **C** Unconstrained PCoA with Bray–Curtis distance showing the root endophytic microbiota of the five plant species. Ellipses cover 68% of data for each plant species. **D**, **E** Shannon (**D**) and Chao 1 (**E**) indexes of root-associated bacteria for the five plant species. The horizontal bars within boxes represent medians. The boxes represent the 75th (top) and 25.th (bottom) percentiles. The upper and lower whiskers extend to data no more than 1.5 × the interquartile range from the upper and lower edges of the box, respectively. HR: healthy root; HS: healthy rhizosphere soil; PR nematode-parasitized root (non-gall part); PS: Rhizosphere soil with RKN; GR, nematode-parasitized root (gall)

To evaluate the effect of RKN parasitism on root-associated microbiota, rhizosphere soil and root samples from five different host plant species, including healthy and RKN-parasitized plants, were collected and analyzed using 16S rRNA gene amplicon sequences (Additional Table S1). Significant differences were found in bacterial community composition between the rhizosphere soil and the root compartments, across all five host plant species (Fig. 1A; Additional Fig. S1A-1B; soil *vs* plant root, adonis: F = 27.39, *R*^*2*^ = 0.27, *p* < 0.001; soil *vs* plant root, ANOSIM: R = 0.92, *p* < 0.001). Bray–Curtis-based principal coordinate analysis (PCoA) showed that the microbial communities were largely grouped according to their ecological niches (soil *vs*. root) (Fig. 1B). Of the 46 bacterial phyla detected, the relative abundances of 35 were significantly different between the rhizosphere soil and the root endophytic compartments (Wilcoxon rank sum test, FDR-adjusted *p* < 0.05; Additional Table S2-1 and S2-2). Overall, Actinobacteria and Acidobacteria were respectively the most significantly enriched in the root endosphere and in the rhizosphere soil. Specifically, niche had a significant effect on both the bacterial diversity (Shannon index: *p* < 0.001) and OTU richness (Chao1 index: *p* < 0.001) between rhizosphere soil and root endosphere samples (Fig. 1D,E).

In addition to the differences observed between rhizosphere soil and root endosphere samples, Canonical Analysis of Principal Coordinates (CAP analysis) also revealed that both plant species and nematode parasitism had prominent effects on bacterial community composition of root-associated microbiota, in the five investigated plant species (soil *vs* root, F = 15.00, *p* < 0.001; healthy *vs* parasitized, F = 15.71, *p* < 0.001; plant species, F = 2.74, *p* < 0.001). Both PCoA and hierarchical cluster analyses indicated notable effects of host plant species on their root-associated bacteria (Fig. 1B; Additional Fig. S1B). Similarly, OTU-based alpha diversity analysis showed that plant species also had a significant effect on both the OTU richness (soil: *p* < 0.001; root: *p* < 0.05) and the bacterial diversity (soil: *p* < 0.05; root: *p* < 0.05) within both the rhizosphere and root endophytic microbiota.

In CAP analysis, ecological niche, host plant species, and nematode parasitism status explained a combined 38.22% of the total variance in root-associated microbiota. Of these three factors, ecological niche and nematode parasitism status explained 14.93% and 13.15% of the total variance, respectively (*p* = 0.002 in both cases), and together with their interaction effect, accounted for 31.17% of the total variance in root-associated microbiota (*p* = 0.002 < 0.01). Further comparisons indicated that the effect of nematode parasitism on bacteria greatly differed between rhizosphere soil and root endosphere. In the rhizosphere soil, there was no significant difference in the observed microbiota between soils associated with healthy plants and those associated with nematode-parasitized plants (Fig. 1A, D, E; healthy *vs* disease soils, adonis: F = 0.498, *R*^*2*^ = 0.017, *p* = 0.837; ANOSIM: R =  − 0.047, *p* = 0.887). Similarly, no difference was found between the healthy and nematode-parasitized root samples in the overall root endophytic microbiota when all five plant species were analyzed together (Fig. 1C–E; healthy *vs* disease roots, adonis: F = 0.848, *R*^*2*^ = 0.039, *p* = 0.635; ANOSIM: R = -0.037, *p* = 0.875). However, when confounding factors of ecological niche and plant species were excluded, the Bray–Curtis-based principal coordinate analysis (PCoA) performed for each plant species showed that the three root tissue samples were significantly different from each other. Specifically, for each plant species, the separation of the three root tissues: healthy roots (HR), gall (parasitized and galled root, GR), and parasitized but non-galled root (PR) explained at least 40% of the total variance of the root endophytic microbiota within individual host species (Additional Fig. S2A-E). Moreover, endophytic bacterial communities of the healthy and parasitized roots (including both the non-galled and the gall tissues) demonstrated significant difference when the effect of RKN parasitism was separately investigated for four of the five plant species: tomato (adonis: F = 2.67, *R*^*2*^ = 0.47, *p* < 0.05; ANOSIM: R = 0.51, *p* < 0.05), lettuce (adonis: F = 4.33, *R*^*2*^ = 0.59, *p* < 0.01; ANOSIM: R = 0.72, *p* < 0.01), snakegourd fruit (adonis: F = 7.57, *R*^*2*^ = 0.72, *p* < 0.01; ANOSIM: R = 0.76, *p* < 0.01), and citrus (adonis: F = 2.97, *R*^*2*^ = 0.50, *p* < 0.01; ANOSIM: R = 0.37, *p* < 0.05). However, no significant difference was found between healthy and nematode-parasitized root endophytic bacteria in celery (Additional Fig. S2E). Interestingly, unlike in the other four plant species, only slight root gall symptom was observed in the fibrous root of celery (data not shown). Different from the endosphere samples, no statistically significant difference was detected between the rhizosphere soil microbiota from the healthy and nematode-parasitized plants, for each of the five plant species.

### Community composition and variation of root-associated bacteria during tomato growth and nematode development

**Fig. 2:**
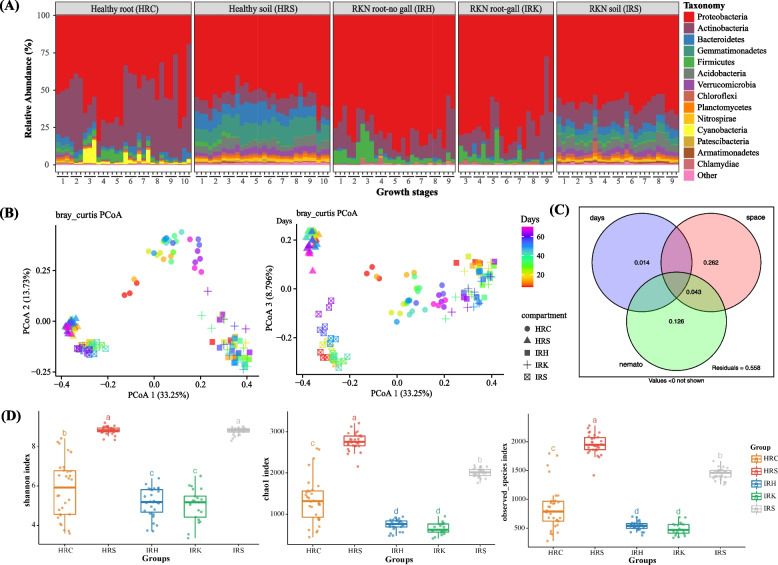
Community composition and variation of root-associated bacteria. **A** Top-15 phylum-level community composition and relative abundance of root-associated bacteria at different growth stages of tomato (see “Methods” for growth stage specifications). **B** Unconstrained PCoA with Bray–Curtis distance showing the root-associated microbiota in healthy and RKN-parasitized samples at different growth stages of tomato plants. **C** Venn diagram for variance partitioning analysis (VPA) showing the variation (adjusted R^2^) contributed by variables “growth days,” “niche” (soil and root), and “nemato” (healthy and nematode-parasitized). Only the factors with *p* < 0.05 were used in the analysis. **D** Shannon, Chao 1 and observed species indexes of root-associated bacteria sampled in relation to growth and nematode development in tomato. HRC: healthy tomato root; HRS: healthy tomato rhizosphere soil; IRH: non-gall parts of the nematode-parasitized root; IRK: galls of the nematode-parasitized tomato root; IRS: Rhizosphere soil with RKN

To specifically differentiate the effect of *Meloidogyne* spp. parasitism on plant root-associated bacteria, we systemically investigated the rhizosphere and the root endophytic microbial community structure, dynamics, and assembly process, at different growth and nematode developmental stages, using tomato plant as a model species (Additional Table S3). Consistent with previous results from the community analysis in five plant species, the soil and root compartments differed significantly in their microbiota (Fig. 2A,B; adonis: F = 56.45, *R*^*2*^ = 0.30, *p* < 0.001; ANOSIM: R = 0.73, *p* < 0.001). The tomato root endophytic microbiota was mainly dominated by phyla such as Proteobacteria, Actinobacteria, and Firmicutes. In the rhizosphere microbiota, Proteobacteria, Actinobacteria, Gemmatimonadetes, Bacteroidetes, and Acidobacteria were instead the main phyla (Fig. 2A). Specifically, OTUs in Orders Rhizobiales, Streptomycetales, Enterobacteriales, Micrococcales, Corynebacteriales, Bacillales, and Pseudomonadales were present, in significantly higher proportions in the root tissues than in the rhizosphere soil (Wilcoxon rank sum test, FDR-adjusted *p* < 0.05; Additional Table S4). Principal coordinate analysis (PCoA) of Bray–Curtis distances revealed that the separation between the rhizosphere soil and root endosphere explained the largest proportion of variance in bacterial communities of root-associated microbiota among tomato plant growth stages and among nematode parasitism development stages (Fig. 2B). Statistical analysis further revealed significant differences between the rhizosphere soil and the root endophytic microbiota in both the richness (Chao1 index: *p* < 0.001; observed_species: *p* < 0.001) and diversity (Shannon index: *p* < 0.001) of OTUs (Fig. 2D).

The PCoA results also showed dissimilarity between root endophytic communities in the healthy and the nematode-parasitized plants (Fig. 2B). The rhizosphere soil samples from healthy (HRS) and parasitized (IRS) plants and the root endophytic (HRC, and IRH, IRK) compartments showed microbiota separate along the PCoA axes (Fig. 2B). In addition, RKN parasitism in tomato plants resulted in significant differentiation of the root endophyte microbial community composition and structure across the examined growth stages, when compared to healthy roots (Fig. 2B; HRC *vs* IRH, adonis: F = 24.27, *R*^*2*^ = 0.31, *p* < 0.001; ANOSIM: R = 0.87, *p* < 0.001; HRC *vs* IRK, adonis: F = 16.83, *R*^*2*^ = 0.26, *p* < 0.001; ANOSIM: R = 0.77, *p* < 0.001). A significant differentiation of bacterial communities over time was also found between the healthy and parasitized soil samples (HRS *vs* IRS, adonis: F = 48.65, *R*^*2*^ = 0.47, *p* < 0.001; ANOSIM: R = 0.99, *p* < 0.001). Variance partitioning analysis (VPA) was used to quantify the contributions of soil/root compartments (niche), RKN parasitism (nemato), and developmental stages (days) to variations in tomato root microbiota (Fig. 2C). Venn diagram showed that soil/root compartments alone explained 26.20% of the total variation in community composition, whereas nematode parasitism explained 12.60% of the variance. Interaction between soil/root compartments and nematode parasitism together explained an additional 4.30% of the variation (Fig. 2C). In total, soil/root compartments, nematode parasitism, and development stages together explained 44.50% of the total variance (Fig. 2C). The results supported the previous findings in microbial community analysis of the five plant species, indicating largely different effect of RKN parasitism on the rhizosphere and root endophytic microbiota. Nematode parasitism had a greater effect on alpha diversity of root endophytic microbiota than on the rhizosphere bacteria (Fig. 2D). Nematode parasitism, however, resulted in a marked decline in OTU richness in both rhizosphere soil and root endophytic microbiota (Fig. 2D; HRC *vs* IRH and IRK,* p* < 0.05; HRS *vs* IRS,* p* < 0.05).

In addition, the community analysis of tomato root-associated bacteria clearly showed that communities varied dramatically across different developmental stages, in each treatment (Fig. 2A; Additional Fig. S3 and S4). In total, bacterial communities became increasingly dissimilar with increasing gap in sampling dates for both the root endophytic and the rhizosphere soil microbiota. Moreover, the dissimilarities of the root endophytic microbiota increased faster than in the rhizosphere soil, as the time distance became greater (Additional Fig. S4A-B). See more details about community variation across different developmental stages in Supporting text (Additional files).

RKN parasitism clearly affected the variation patterns of the endophytic microbiota along the host plant growth stages. For example, the community dissimilarities along time in healthy plants were greater than those in nematode-parasitized ones (IRH and IRK), with time explaining a greater proportion of endophytic community variation in healthy than in parasitized samples (Additional Fig. S4A; HRC: *R*^2^ = 0.35; IRH: *R*^2^ = 0.19; IRK: *R*^2^ = 0.11), especially for the gall-associated microbiota (IRK). Further pairwise analysis for the variations of microbial diversity along the time gradients using the Shannon, Chao1, and observed species indexes also showed that both the plant developmental stages and nematode parasitism status significantly affected the root endophytic taxa. The establishment of endophytic species in healthy tomato roots over time showed a gradual decrease of species richness and bacterial diversity, whereas nematode parasitism resulted in relatively minor changes of community dissimilarity in the endophytic species in parasitized roots (Additional Fig. S4C; Shannon: HRC *p* < 0.001, IRH *p* = 0.123, IRK *p* = 0.835; Chao1: HRC *p* < 0.001, IRH *p* = 0.001, IRK *p* = 0.024). Interestingly, rhizobacterial species richness and diversity underwent relatively minor changes with a more phylogenetically diverse set of microbiota than those of root endophytes, across different developmental stages (Fig. 2D; Additional Fig. S4C; Shannon: HRC *p* < 0.001, HRS *p* = 0.312; Chao1: HRC *p* < 0.001, HRS *p* = 0.063).

### Effects of RKN on microbial community assembly in tomato plants

**Fig. 3:**
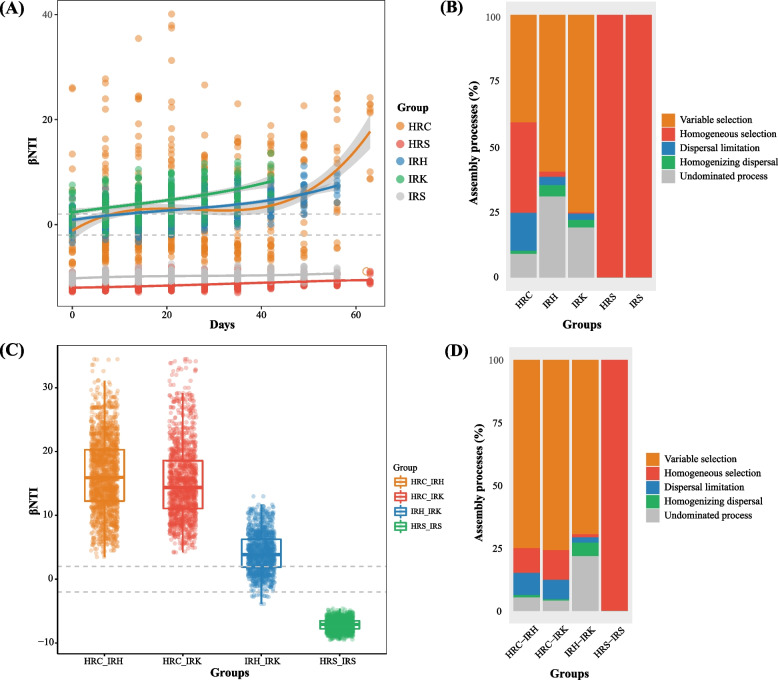
Bacterial community assembly processes of tomato root-associated taxa in relation to plant growth and nematode parasitism development. **A** Relationships between the values of the weighted beta nearest taxon index (βNTI) and day distances inferring the changes in time of deterministic/stochastic assembly processes (HRC: *R*^*2*^ = 0.10, *p* < 0.001; HRS: *R*^*2*^ = 0.20, *p* < 0.001; IRH: *R*^*2*^ = 0.27, *p* < 0.001; IRK: *R*^*2*^ = 0.22, *p* < 0.001; IRS: *R*^*2*^ = 0.08, *p* < 0.001). Horizontal dashed gray lines indicate upper and lower significance thresholds at βNTI < 2 and > 2, respectively. **B** Percentage of turnover in community assembly of root-associated taxa indicating the relative contribution of deterministic (homogeneous and variable selection), stochastic (dispersal limitation and homogenizing dispersal), or undominated processes. **C** βNTI values for pairwise community comparisons between samples with healthy and RKN parasitism conditions. **D** Percentage of the pairwise community turnover indicating the relative contribution of deterministic, stochastic, or undominated processes in driving the turnover of bacterial communities from the healthy to RKN parasitism conditions of roots. For descriptions of sample groups, please see Fig. 2

To estimate the community assembly and ecological process of root-associated bacteria in healthy and nematode-parasitized tomato plants over time, the β-nearest taxon indices (βNTI) of all samples were calculated (Fig. 3). The relationships between βNTI and the sampling date distance indicated that community dissimilarity of root-associated microbiota in each treatment was significantly correlated with the distance between sampling dates (*p* < 0.001; Fig. 3A). βNTI values of the paired time plots for each treatment were below − 2 and above + 2 (|βNTI|> 2), indicating that community assembly and variation of tomato root-associated taxa along the time distance in each treatment were dominated by deterministic processes (Fig. 3A). Random factor only played a dominant role in a few plots at a short sampling date distance in the healthy root endophytic bacteria (|βNTI|< 2). At an increased time distance between sampling dates, the community dissimilarity between pair of plots significantly increased, in both the rhizosphere and root endophytic microbiota (Fig. 3A). Community variation and dissimilarity of endophytic taxa along time distance in the parasitized roots showed a pattern different from those of healthy tomato roots. Moreover, the microbial community assembly in galls (IRK) appeared mostly affected by deterministic processes. This was consistent with community variation and dissimilarity of the healthy root endophytic microbiota (HRC) being greater than those of nematode-parasitized roots (IRH and IRK) (βNTI > 2; Fig. 3A). Under the influence of RKN, the bacterial community structures along the time distance were driven mostly by variable selection pressures (IRH, 59.54%; IRK, 75.24%). In contrast, the community compositions of samples from healthy plants were mostly structured by variable selection (40.92%), homogeneous selection (34.48%), and dispersal limitation (14.48%) (Fig. 3B). The higher proportion of variable selection in the nematode-parasitized root endophytic microbiota (IRH and IRK) over those of healthy root samples (HRC) suggested that parasitism resulted in a shift in selective pressure and bacterial movements within the root endophere (Fig. 3B) [34]. Compared with the dominance of variable selection on endophytic microbiota (βNTI > 2), the bacterial communities of both healthy and nematode-parasitized soil samples were more impacted by homogeneous selection (βNTI <  − 2) (Fig. 3A,B). The dominance of homogeneous selection in the rhizosphere taxa (HRS and IRS) suggested that community differences of the soil samples along time were mainly caused by the relatively stable selective pressure in the soil environments [34].

The βNTI values for all pairwise community comparisons between treatments helped reveal the assembly process and community variation during the turnover from a healthy to a parasitized status (Fig. 3C). Specifically, pairwise community turnover in root samples HRC-IRH and HRC-IRK differed from that in IRH-IRK (Fig. 3C). Deterministic processes (βNTI > 2) dominated the endophytic community turnover of the pairwise comparisons HRC-IRH and HRC-IRK (Fig. 3C). In contrast, pairwise community turnover in rhizosphere soil samples HRS-IRS was also dominated by deterministic processes but with βNTI values below − 2 (|βNTI|> 2; Fig. 3C). In the pairwise community turnover from healthy to parasitized samples, variable selection due to deterministic processes (> 75%) played a more pronounced role in the bacterial assembly process of samples HRC-IRH and HRC-IRK than for IRH-IRK (Fig. 3D). The pairwise community turnover of IRH-IRK was mainly derived by the variable selection (69.33%) and undominated process (21.72%) (Fig. 3D). The pairwise community analysis between healthy and parasitized root samples illustrated the effect of nematode parasitism on the community assembly and variation of root endophytic microbiota. Compared with the healthy tomato root samples (HRC), the community structure and variation of the nematode-parasitized root endophytic taxa (IRH and IRK) showed a major effect from RKN-caused environmental changes. Furthermore, compared with the parasitized root samples (IRH), the shift of selective pressure and organism movement resulting from RKN parasitism likely played a dominant role in shaping the gall-associated endophytic microbiota (IRK). The ecological process analysis for the tomato root endophytic microbiota indicated that community assembly and variation of the endophytic taxa in the parasitized tomato roots were inevitably correlated with presence of RKN. In contrast, nematode parasitism had less effect on microbial community differences in the rhizosphere soil microbiota, which was largely driven by homogeneous selection (Fig. 3C,D).

### Biomarkers and key bacterial taxa associated with RKN parasitism in tomato roots

**Fig. 4:**
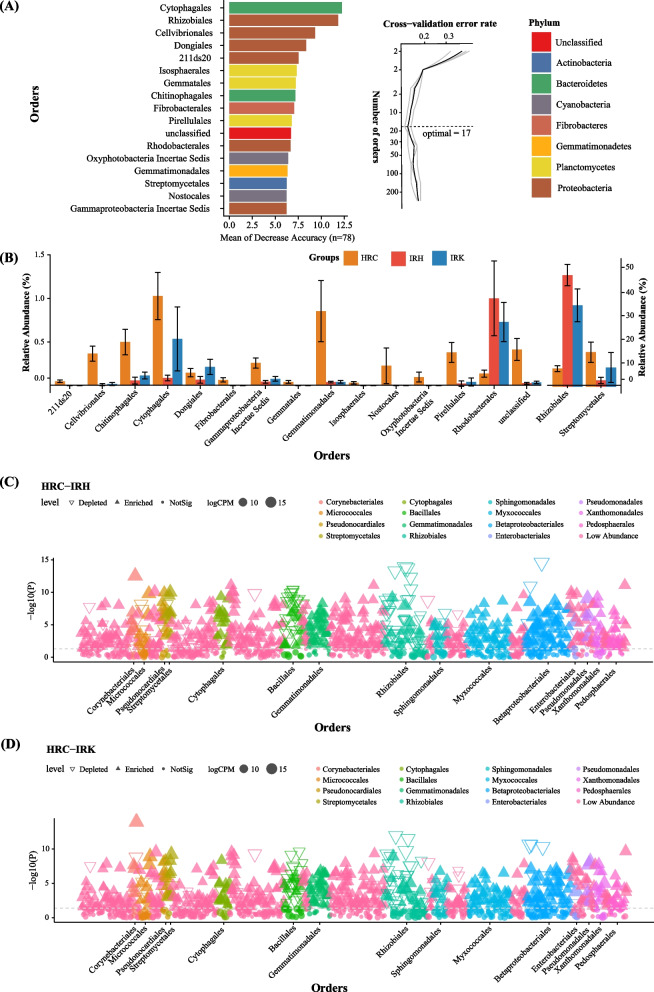
Identification of biomarkers and key taxa to differentiate the healthy and RKN-associated root endophytic bacteria.** A** Top 17 bacterial orders identified to discriminate the relative abundance of endophytic microbiota in the RKN-parasitized root samples (IRH and IRK) *vs* healthy tomato roots, as shown by the Random Forest method. Biomarker taxa were ranked in descending order of importance for model accuracy. **B** Order-level biomarkers with higher relative abundance in the endophytic microbiota of healthy and parasitized tomato plants (bars = means; error bars = SE). **C**, **D** Manhattan plots showing OTUs depleted or enriched in the healthy samples *vs* parasitized samples IRH (**C**, non-gall part of root) or IRK (**D**, galls). Each dot or triangle represents a single OTU. OTUs enriched or depleted in the healthy tomato root are represented by filled or empty triangles, respectively (Wilcoxon rank sum test, FDR-adjusted *p* < 0.05). OTUs are arranged and colored according to the bacterial order. For descriptions of sample groups, see Fig. 2

As demonstrated above, RKN parasitism had more significant effects on the root endophytic microbiota than on the rhizosphere soil taxa. As RKN complete most of their life cycle inside host roots, we investigated the occurrence of bacteria specifically associated with parasitism that could differentiate healthy and nematode-parasitized roots, using a Random Forest method [16, 17, 21]. Our analyses indicated that the cross-validation error curve was stabilized when 17 most relevant bacterial orders were used to define putative biomarker taxa (Fig. 4A). Of these, 15 orders showed higher relative abundance in healthy (HRC) than in parasitized roots (IRH and IRK). The remaining two orders, Rhizobiales and Rhodobacterales, showed higher relative abundance in nematode-parasitized roots than in healthy samples (*p* < 0.05; Fig. 4B). Rhizobiales and Rhodobacterales contain biological nitrogen fixers. They were enriched in the nematode-parasitized tomato plants, an observation consistent with previous results showing nitrogen-fixing Rhodocyclales being highly enriched in the gall-associated microbiota. The results suggest that these biological nitrogen fixers might serve as key biomarker taxa to differentiate nematode-parasitized plants from healthy ones [22].

To identify the differences in the root microbiota between healthy and nematode-parasitized tomato plants, we examined the enriched and depleted OTUs in RKN-parasitized plants (IRH and IRK) with respect to the healthy roots (HRC), according to their taxonomy (Wilcoxon rank sum test, FDR-adjusted *p* < 0.05). Manhattan plot analysis indicated a total of 131 OTUs (accounting for 69.80% of total sequencing reads) enriched in the IRH samples. These taxa mainly belonged to Rhizobiales (49 OTUs, 44.99%), Betaproteobacteriales (2 OTUs, 11.63%), and Firmicutes (27 OTUs, 3.25%) (Fig. 4C, Additional Table S4-2 and S5-1). The results for the enriched or depleted OTUs were consistently obtained in the IRK *vs* healthy samples (HRC) (Fig. 4C,D, Additional Fig. S5A-B and Additional Table S4-2 and S4-3, S5-1 and S5-2). The enriched taxa (118 OTUs, accounting for 66.10% of total sequencing reads) in the gall-associated microbiota (IRK) mainly belonged to Rhizobiales (36 OTUs, 31.41%), Betaproteobacteria (1 OTU, 14.46%), and Firmicutes (21 OTUs, 1.36%) (Fig. 4D, Additional Table S4-3 and S5-2). Specifically, the Rhizobiales OTUs enriched in the gall-associated microbiota (IRK) occurred in most nematode-parasitized root samples, and in high abundance during late parasitism stages (Additional Fig. S3A). OTUs belonging to Firmicutes were mainly enriched in early nematode parasitism stages, with some of them reduced during subsequent stages (Additional Fig. S3A). In contrast, in healthy plant samples, OTUs belonging to a wide range of taxa, including Actinobacteria, Xanthomonadales, Pseudanabaenales, Enterobacteriales, Flavobacteriales, and Solibacterales were enriched (Fig. 4C,D, Additional Fig. S5A-B, and Additional Table S5-1 and S5-2). The enriched Rhizobiales OTUs in the parasitized roots (IRH and IRK) belonged to the genus *Agrobacterium* (*Agrobacterium radiobacter*) and several known nitrogen-fixing bacterial genera such as *Rhizobium* and *Bradyrhizobium* (Additional Table S5-1 and S5-2). The highly enriched OTUs in Burkholderiaceae such as *Acidovorax* and *Ralstonia* contained taxa known to be pathogenic to plants (Additional Fig. S5C-D, and Additional Table S5-1 and S5-2).

### Prediction and exploration of functional attributes of root endophytic bacteria in RKN parasitism of tomato

**Fig. 5:**
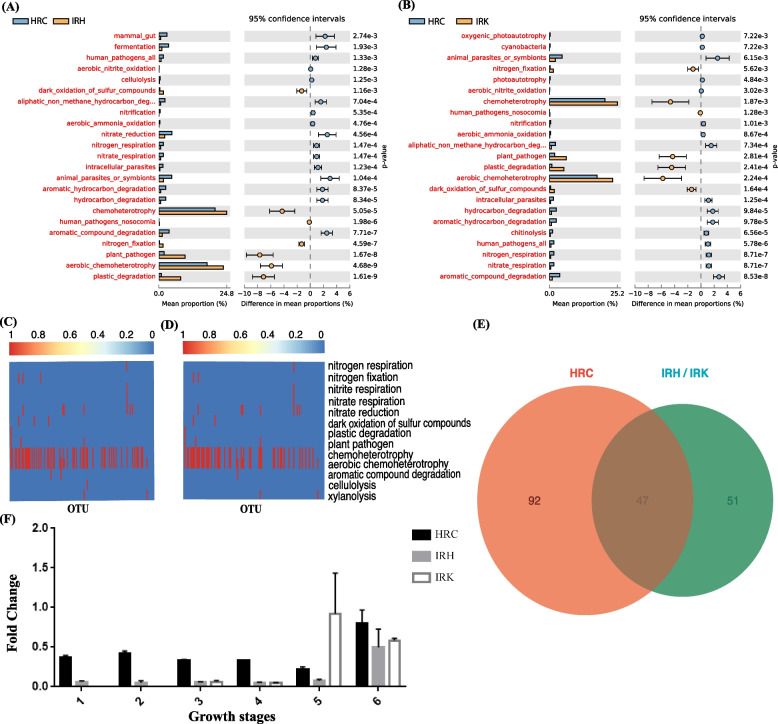
Functional attribution and association of root endophytic bacterial taxa to RKN parasitism in tomato plants. **A**, **B** Significantly enriched functional pathways of tomato root endophytic bacterial taxa in healthy roots against RKN-parasitized samples IRH (**A**, non-gall part of disease root) or IRK (**B**, gall), based on FAPROTAX (*p* < 0.05). **C**, **D** Heatmaps showing the FAPROTAX-based functional pathways with higher relative abundance of OTUs enriched in non-gall part of root (**C**, IRH) or gall (**D**, IRK). Each row represents an OTU. The presence of functions is shown in red. **E** Venn diagram shows the overlapping of the identified proteins in healthy (HRC) and RKN-parasitized root samples (IRH and IRK) using metaproteomic data. **F** Quantitative analysis for the nitrogenase gene (nifH) of healthy and RKN-parasitized root samples at different growth and disease developmental stages, using quantitative PCR (qPCR). For descriptions of sample groups, see Fig. 2

To reveal the potential functional attributes of root endophytic microbial communities related to RKN parasitism, we annotated the functions of OTUs using FAPROTAX [35, 36]. The analyzed OTUs included bacteria from the tomato rhizosphere and the tomato root endosphere at different developmental stages. Seven specific pathways, including chemoheterotrophy, aerobic chemoheterotrophy, plant pathogen, plastic degradation, dark oxidation of sulfur compounds, and nitrogen fixation, were significantly enriched in the root endophytic microbiota of nematode-parasitized tomato plants (IRK and IRH), compared to the healthy ones (HRC) (*p* < 0.01; Fig. 5A,B; Additional Table S6-1 and S6-2). We specifically paid attention to the enriched function in nitrogen fixation, which corresponded to enrichment of nitrogen-fixing bacteria found in nematode-parasitized samples (IRH and IRK) in the previous community taxonomic analysis (Fig. 4C,D, and Additional Table S5). In contrast, other nitrogen cycling functional groups, i.e., aerobic ammonia oxidation, nitrification, aerobic nitrite oxidation, nitrate respiration, nitrogen respiration, and nitrate reduction, showed higher relative abundance in the healthy (HRC) tomato root samples than in the parasitized ones. The results suggest the importance of nitrogen availability and nitrogen fixation to nematode parasitism in tomato roots (*p* < 0.01; Fig. 5A,B; Additional Table S6-1 and S6-2). Altogether, 36 OTUs related to nitrogen fixation, mainly belonging to Rhizobiales, Azospirillales, and Rhodospirillales, were identified in tomato root-associated taxa. In addition, 6 identified OTUs, mainly belonging to Rhizobiales (*Agrobacterium*) and Betaproteobacteriales (*Ralstonia*), were related to plant pathogens (Additional Table S7). The cumulative abundance of nitrogen fixation-related OTUs enriched in IRK (4 OTUs) and IRH (4 OTUs) was 3.67 and 3.88%, respectively. The cumulative abundance of plant pathogen-related OTUs enriched in IRK (4 OTUs) and IRH (3 OTUs) was 24.77 and 15.65% respectively (Additional Table S7). Of special interest was OTU_2, taxonomically defined as *Agrobacterium radiobacter*, that was the most common OTU enriched in the RKN-parasitized samples (21.87% in IRH and 13.64% in IRK). Being classified into genus *Agrobacterium*, OUT_2 was originally grouped into pathogenic bacteria in FAPROTAX-based analysis. However, *A. radiobacter* is known as a non-pathogenic species, with nitrogen-fixing ability and found primarily as an endophyte.

Most identified OTUs related to nitrogen fixation and plant pathogenesis were also included in the enriched bacterial communities (Rhizobiales and Betaproteobacteriales) of the RKN-parasitized samples IRK and IRH (Fig. 4C,D; Additional Table S5 and S7). Therefore, we also collected and annotated the specifically enriched OTUs using FAPROTAX. Notably, the enriched OTUs in the parasitized samples IRK (118 OTUs) and IRH (131 OTUs) were mainly related to the enriched pathways described previously, including chemoheterotrophy, aerobic chemoheterotrophy, plant pathogen, plastic degradation, dark oxidation of sulfur compounds, and nitrogen fixation (Fig. 5C,D). The results suggested that RKN parasitism of tomato roots was accompanied by a specific enrichment and high abundance of nitrogen-fixing bacteria and pathogenic bacterial complexes. Taken together, both the OTU enrichment analysis and the functional comparisons for the root endophytic microbiota between healthy and nematode-parasitized plants showed a taxonomic and functional association of endophytic nitrogen-fixing bacteria with nematode parasitism in tomato plants.

### Effect of nitrogen-fixing bacteria on parasitism in tomato plants

To validate the functional and taxonomic associations of nitrogen-fixing bacteria to RKN parasitism, a comparative metaproteomic analysis was conducted for the root endophytic samples collected from four different developmental stages of healthy and parasitized tomato roots. In total, 6787 and 7162 proteins were separately identified from the healthy (HRE 1–4) and the RKN-parasitized tomato roots, respectively (galls: NKRE 1–4; and non-gall tissues: NKHRE 1–4). Among them, only 346 (HRE) and 150 (NKRE and NKHRE) proteins belonged to bacteria, suggesting a lower representation of bacterial protein contents in plant root tissues even after the enrichment of microbial cells by filtering out plant host tissues. Among these bacterial proteins, 47 were common to all three sample groups. Fifty-one quantifiable proteins were specifically associated with RKN-parasitized samples (NKRE and NKHRE) (Fig. 5E; Additional Table S8). Interestingly, the key nitrogenase iron protein (NifH) involved in biological nitrogen fixation was identified throughout the nematode parasitism stages in tomato roots (NKRE 1–4 and NKHRE 1–4) whereas it was not identified in the healthy tomato root samples (Additional Table S8). In addition, a specific protein (NolF) involved in root nodulation was also identified in the RKN-parasitized samples (Additional Table S8). Consistent with the above functional predictions, the metaproteomic data from root endophytic microbiota of the healthy and parasitized tomato samples substantiated the hypothesis that nitrogen-fixing bacteria are involved in tomato root parasitism by RKN.

Furthermore, we quantified the relative copy numbers of the nitrogenase gene (*nifH*) in the healthy and nematode-parasitized root samples with qPCR. The results showed that *nifH* was widely distributed in the tomato root endophytic microbiota. In healthy plant root tissues, *nifH* was relatively abundant during early plant development, but decreased during later stages (Fig. 5F). In contrast, the copy number of *nifH* gene dramatically increased by more than ten-folds in the root gall microbiota of parasitized tomato plants (IRK) at the fifth sampling (50 days), consistent with its significant enrichment with the nitrogen-fixing bacteria in the gall (Fig. 5F). The result showed that nitrogen-fixing bacteria were common in the root endophytic microbiota in tomato plants. However, the identification of the nitrogenase protein NifH in nematode-parasitized tomato roots but not in healthy root samples indicates higher abundances of nitrogenase gene and nitrogen-fixing bacteria in the galls (IRK) of parasitized plants.

**Fig. 6:**
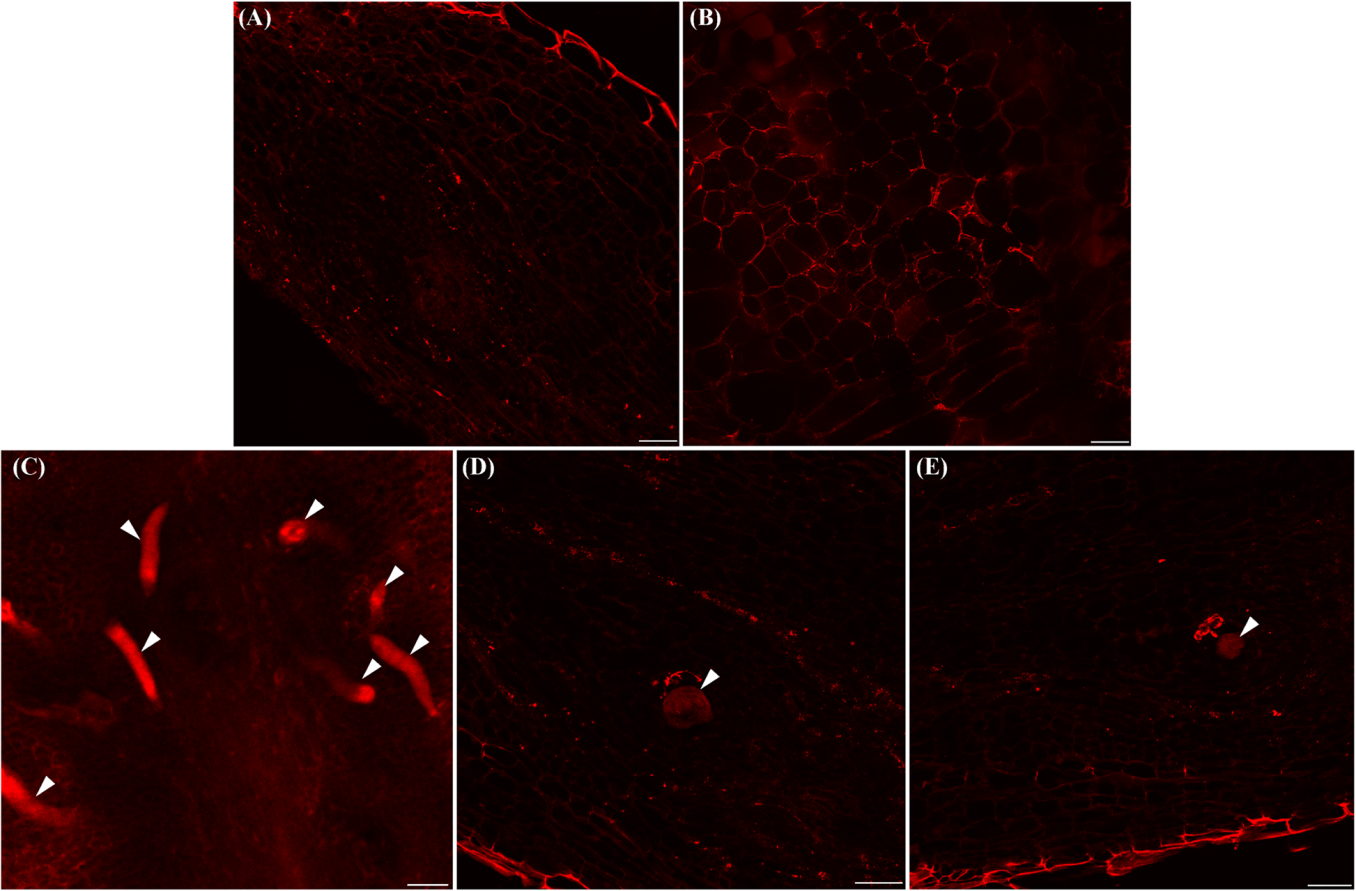
Visualization of nitrogen-fixing bacteria and root-knot nematodes in tomato roots. **A**, **B** FISH protocol fluorescence signals indicated the distribution of nitrogen-fixing bacteria in tomato root tissues by labeling the nitrogenase gene (nifH). **C–E** Fluorescence signals from nitrogen-fixing bacteria associated with root-knot nematode juveniles (**C**) or adult females (**D**, **E**) using the nifH probe in tomato root tissues. Arrows indicated nematode juveniles (**C**) or female adults (**D**, **E**). Scale bar = 100 μm

To visualize the nitrogen-fixing bacteria associated with RKN during parasitism in tomato roots, a probe specific for *nifH* was used to detect these bacteria through fluorescence in situ hybridization (FISH) (Fig. 6A–E). Negative control showed no fluorescence as tomato root tissues was treated according to the standard FISH protocol but without the *nifH* gene probe. Clear fluorescence signals corresponding to *nifH* were found in the epidermis of tomato root, and with the spot-like signals in the cortex or inside the root tissues (Fig. 6A,B). Although thick plant cell layers weakened the signals, clear fluorescence were particularly found in structures with a shape similar to nematode juveniles or female adults in root and gall (Fig. 6C–E). Compared with the spot-like fluorescence signals in the root tissues, the brighter *nifH* fluorescence in nematode J2 or female adults in root galls than in other parts of the root suggests a putative tight RKN association with nitrogen-fixing bacteria in parasitized roots (Fig. 6A–F).

### Elevated nitrogen level suppressed RKN parasitism in tomato plants in pot experiments

To explore the association between RKN parasitism and endophytic nitrogen-fixing bacteria, control experiments were performed by applying various nitrogen sources to tomato plants grown in nematode-parasitized soils. Altogether, 13 types of nitrogen-rich or nitrogenous compounds, including organic and inorganic nitrogen fertilizers, chicken manure, and chicken manure-based biofertilizer, were applied to the nematode-parasitized soils in pot experiments (Additional Table S9). The addition of external nitrogen to soil significantly influenced the occurrence of RKN in tomato roots (Additional Table S9). Specifically, 10 of the 13 nitrogen sources showed a significant gall suppression effect (Additional Table S9; *n* ≥ 3, *p* < 0.05). In contrast, the application of NH_4_Cl, (NH_4_)_2_CO_3_ and NH_3_·H_2_O increased root gall numbers in tomato roots (Additional Table S9).

**Fig. 7:**
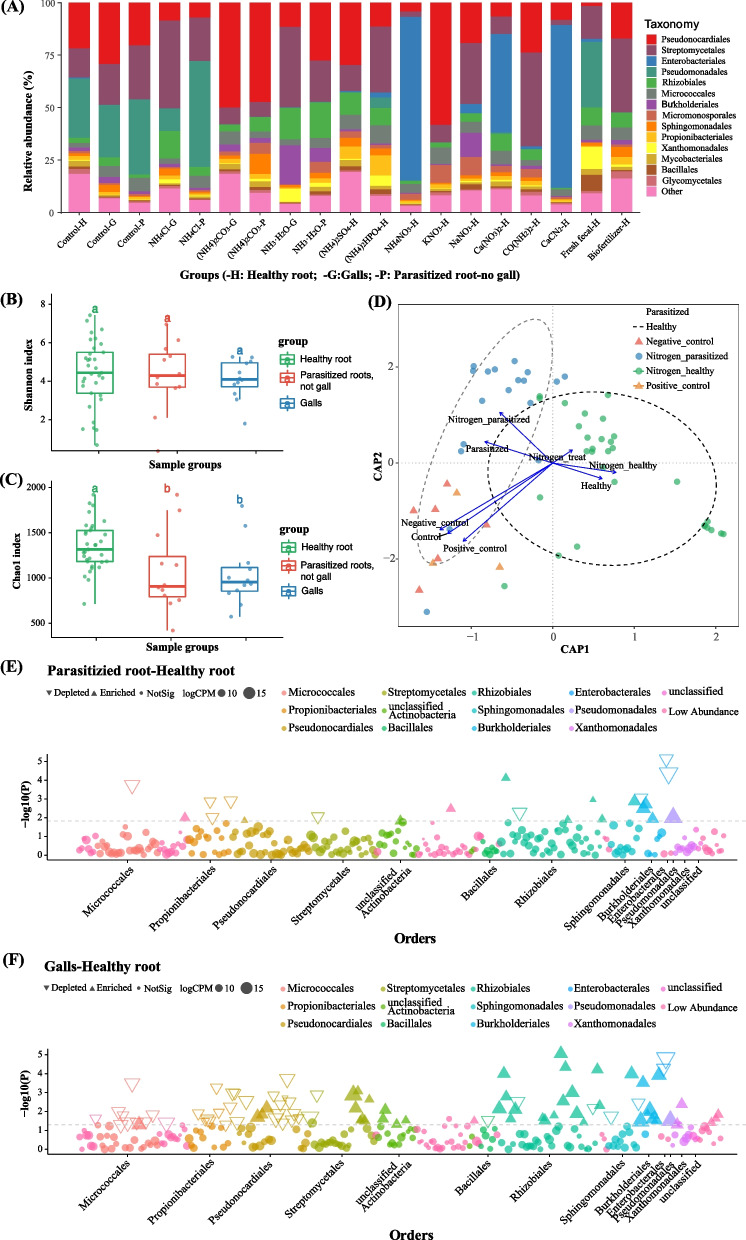
Root endophytic microbiota of tomato plants grown in soils with root-knot nematodes, amended with different nitrogen sources.** A** Order-level endophytic community distribution of tomato plants grown in soils with *Meloidogyne* spp., amended with different nitrogen sources. See Table S9 for treatment description. **B**, **C** Shannon and Chao 1 indexes of root endophytic bacteria in tomato plants supplied with different nitrogen sources. **D** CAP showing similarity and groups of healthy and RKN-parasitized root samples collected from tomato plants treated with the nitrogen sources. **E**, **F** Manhattan plots showing OTUs depleted or enriched in parasitized samples with *Meloidogyne* spp. (**E**, non-gall part of parasitized root; **F**, galls) *vs* healthy samples. OTUs are arranged and colored according to the bacterial order

To reveal the mechanism underlying the nitrogen form effect on RKN, we compared the root endophytic microbiota between healthy and nematode-parasitized tomato samples (Fig. 7A–C). The healthy tomato plants included both the healthy control and the tomato plants grown in the soils amended with 10 nitrogen sources where RKN parasitism was significantly reduced (3 replicates each). In contrast, the parasitized samples included the parasitized control (tomato plants grown in nematode-parasitized soil with no additional nitrogen) and the parasitized tomato plants grown in soils amended with NH_4_Cl, (NH_4_)_2_CO_3_ and NH_3_·H_2_O (3 replicates each) (Additional Table S9). Overall, the microbial community structure and OTU richness of the root endosphere microbiota were significantly different between the healthy and the parasitized plants (Fig. 7A, C; healthy *vs* parasitism: adonis: F = 2.76, *R*^*2*^ = 0.09, *p* < 0.001). However, no significant community difference was found between the healthy control and the healthy tomato plants amended with the 10 nitrogen sources, or between the parasitized control and the parasitized tomato plants amended with the three nitrogen sources. CAP analysis showed that nitrogen amendment and nematode parasitism separated root endophytic taxa of the healthy and the parasitized tomato plants (Fig. 7D; CAP1: *F* = 7.18, *p* < 0.001; CAP2: *F* = 2.26, *p* < 0.05). Nitrogen amendment had a significant effect on the microbial community structure and composition of the healthy tomato plants (Fig. 7D; Additional Table S9). Compared to the healthy samples, the 13 enriched OTUs in the parasitized tomato tissues outside of the gall regions mainly belonged to Pseudomonadales, Burkholderiales (mainly *Ralstonia*), and Rhizobiales, whereas 47 OTUs, mainly belonging to Streptomycetales, Pseudomonadales, Burkholderiales (mainly *Ralstonia*), and Rhizobiales, were enriched in the galls (Fig. 7E,F and Additional Table 10). Consistent with previous results, Rhizobiales OTUs belonging to known nitrogen-fixing bacteria were also enriched in all the parasitized tomato samples, including the parasitized control (i.e., no nitrogen amendment) and the parasitized tomato plants grown in soils amended with NH_4_Cl, (NH_4_)_2_CO_3_ and NH_3_·H_2_O (Fig. 7E,F and Additional Table 10). In addition, more Rhizobiales were enriched in the gall (16 Rhizobiales OTUs, out of the 47 OTUs enriched) than the non-gall part of the parasitized roots (4 Rhizobiales OTUs, out of the 13 OTUs enriched).

## Discussions

RKN cause severe damages to plant health and crop production and are the most important root-damaging plant-parasitic nematodes [15, 18]. Many studies have demonstrated that RKN parasitism can also cause significant changes on the diversity and structure of plant-associated microbial communities, including both the rhizosphere soil and the root endophytic microbiota [22, 26, 28–30]. However, much remains unknown about functional contributions of the microbial communities and how their variation relates to RKN parasitism and plant health. Here, we showed that multiple factors influence variations and differentiations of root-associated microbiota, including differences in ecological niche (rhizosphere soil *vs* root endophytes) and the health status, developmental stage, and species of host plants (Fig. 1 and Fig. 2). RKN showed significant interactions with root-associated microbiota at the level of ecological niche, developmental stage, and plant species.

The effect of RKN parasitism on root-associated microbiota appeared to be highly niche-specific. The rhizosphere soil and root endophytic spaces showed large differences in the community structure and taxa composition in all the examined healthy and nematode-parasitized plants (Fig. 1 and Fig. 2). Moreover, nematode parasitism had a greater effect on root endophytes than on rhizosphere microbiota. The effect of RKN on endophytic taxa was likely related to nematodes completing most of their life cycle inside the roots, potentially linking root endophytic bacteria to RKN parasitism. As shown by the ecological process analysis, the most important change in root endophytes was brought about by the initial nematode invasion, after which the microbiota remained relatively stable. RKN parasitism resulted in a changed root endophytic microenvironment, which likely imposed a selective pressure different from the healthy root environment, leading to a re-structured microbial community within parasitized roots (Fig. 3C,D). Interestingly, more unique microbial taxa were found in the galls than in the gall-free nematode-parasitized tissues. Together, these results suggest that the parasitizing nematodes likely brought their own microbial flora to the root site of parasitism, with a subsequent population diversity expansion. A recent study also demonstrated that the introduction of RKN increased fungal and bacterial alpha diversity of rhizosphere microbiota [37].

Our analyses identified several key bacterial taxa within the tomato root endosphere during RKN parasitism. Comparisons between healthy and nematode-parasitized root samples showed differences in two major functional microbial groups: (i) plant pathogenic bacteria, including some species in genera *Agrobacterium* and *Ralstonia*, and (ii) nitrogen-fixing bacteria, including *A. radiobacter* and species in genera *Rhizobium* and *Bradyrhizobium* (Fig. 4). Correspondingly, two enriched functional pathways, bacterial pathogenesis and biological nitrogen fixation, were identified in the nematode-parasitized tissues. However, the abundance of nitrogen-fixing bacteria and nitrogen fixation in the parasitized samples was likely substantially underestimated in FAPROTAX analysis. OTU_2, which was taxonomically defined as *A. radiobacter*, was known as a non-pathogenic *Agrobacterium* species [38]. Studies showed that *A. radiobacter* was also isolated and identified as a non-symbiotic endophyte and a nitrogen fixer [39–41]. Therefore, *A. radiobacter* was likely the most important biological nitrogen fixer highly enriched in RKN-parasitized tomato plants. The functional involvement of nitrogen fixation to RKN parasitism was further supported by the identifications of high copy number of the *nifH* gene and abundant NifH protein in nematode-parasitized root samples (Fig. 5E,F; Additional Table S8). Furthermore, through FISH using *nifH* as a probe, we observed that nitrogen-fixing bacteria were highly abundant and closely associated with RKN, including parasitized roots and galls. The observation of fluorescence-labeled nitrogen-fixing bacteria within the nematode feeding sites (galls) is consistent with a high copy number of *nifH* gene at these sites (Fig. 5F). Interestingly, the highest fluorescent signal seemed to be associated with RKN juveniles or adult females (Fig. 6). The ecological and reproductive significance of this observation remains to be investigated. Different from nitrogen-fixing bacteria, plant pathogenic bacteria associated with parasitism by RKN have been reported [14, 22, 26]. Together, our report is the first to demonstrate that biological nitrogen fixation is taxonomically and functionally associated with nematode parasitism in plants.

As an essential nutrient required for all living organisms, nitrogen is among the most common limiting nutrients for plants. For RKN, which depend completely on root giant cells as their sole source of nutrients, nitrogen is also a limiting nutrient for growth, development, and reproduction [16, 19]. The nutrient limitation will likely become even more severe for both the nematode and their host plant after the roots are damaged by RKN. For example, one study showed that RKN parasitism affected both nitrogen uptake and distribution during vegetative growth of grapevines [42]. Therefore, the enrichment and occurrence of nitrogen-fixing bacteria in nematode-parasitized roots, especially within the gall, indicated that nematodes interacted with such bacteria, likely to get a benefit from nitrogen fixation [12, 33]. However, the mechanism of such nitrogen transfer during parasitism remains unknown.

In addition to the interaction between nitrogen-fixing bacteria and RKN, studies have demonstrated that both nitrogen-fixing symbiosis and free-living diazotrophic community dynamics are sensitive to soluble nitrogen concentration in the environment [43–46]. Field soils supplemented with nitrogen fertilizers often show significantly reduced biological nitrogen-fixing activities, with a lower number of nitrogen-fixing bacteria in the plant rhizosphere and root endosphere [43–46]. To examine the role of nitrogen-fixing bacteria, we altered the composition of the tomato root endophytic microbiota by adding different nitrogen sources to their soil. The results suggested that the occurrence of RKN in tomato plants was positively associated with the enrichment of the nitrogen-fixing bacteria in the root endophytic microbiota.

The sensitivity of RKNs and parasitism to organic fertilizers and several other nitrogen sources is consistent with nitrogen as a critical factor for their growth and development, and for the occurrence of galls. In fact, using nitrogen management to control plant-parasitic nematodes has been practiced for a long time [47–49]. However, the underlying mechanism about how nitrogen availability affects RKN parasitism is largely unknown. Proposed hypotheses to explain the nitrogen fertilization effects include the following: (i) stimulating growth and trap formation by nematode-trapping fungi, and then supplying roots with the organic matter released by the dead nematodes, and (ii) the attraction or repulsion effects of nitrogen compounds for nematodes, such as NH_4_^+^, NO, or other salt ions [37, 48–51]. An alternative mechanism explaining the biological control effects of soil nitrogen management on RKN involves the growth and activity of nitrogen-fixing bacteria in plants. Such bacteria are sensitive to high soil nitrogen concentrations, which often suppress nodulation and nitrogen-fixing activity [43–46]. A similar interference mechanism may operate for nitrogen-fixing bacteria within RKN galls, where an external nitrogen source may suppress nitrogen-fixing bacteria activity, which subsequently may reduce the growth, development, and/or reproduction of nematodes in roots. The reason for the differential control effects of nitrogen sources or biofertilizers on RKN may be related to other factors, including the form of nitrogen, nitrification and denitrification, plant host species, and abiotic factors such as pH, temperature, oxygen, and phosphorus that may influence nitrogen-fixing bacteria associated with RKN [44–46, 52]. An especially intriguing topic for future research would be to analyze the interactions and nutrient flows among nitrogen fertilizer sources, nitrogen-fixing bacteria, and RKN in nematode-parasitized plants, and how such interactions influence the assembly and activity of nitrogen-fixing bacterial communities in parasitized roots, including galls and root nodules. Understanding and exploring the ecological and evolutionary mechanisms for the assembly of nitrogen-fixing bacteria with nematode parasitism will also help us better reveal parasitic mechanism of RKN, and the complex interactions linking nematodes, plant host, and associated microbiota.

At the molecular and biochemical levels, there are several intriguing questions about the interactions between nitrogen-fixing bacteria with nematode parasitism. For example, how does bacterial nitrogen fixation occur within the nematode feeding sites? Is it different from the free-living and symbiotic nitrogen fixers? And, how is the fixed nitrogen shared among bacteria, nematodes, and potentially host plants? Indeed, although *Rhizobium* spp. are common symbiotic nitrogen-fixing bacteria of legume plants, several non-symbiotic *Rhizobium* lineages have been identified, with many strains in these lineages lacking the ability to form nodule and fix nitrogen [53, 53–55]. Moreover, *A. radiobacter* was the most common bacterium highly enriched in the nematode-parasitized root samples (IRK and IRH) (Additional Table S7). Several *Agrobacterium* species are known biological nitrogen fixers, including *A. radiobacter* [39, 40, 53, 56, 57]. Therefore, *A. radiobacter* likely played a more important role as a biological nitrogen fixer within RKN-parasitizing tomato plants, than the *Rhizobium* species. Several studies reported that the root nodules produced by legume rhizobia and galls caused by RKN parasitism shared certain structural and developmental similarities, with some common regulatory pathways and mechanisms involved in the formation of galls and rhizobia nodules [58–60]. In addition, legumes can obtain their nitrogen through two biological sources, such as their symbiotic nitrogen fixers (the commonly known source) and their nitrogen-fixing and non-nitrogen-fixing endophytes [60–63]. The biologically fixed nitrogen may be directly taken up and distributed to the whole plant or stored in giant root cells for later distribution [58, 61–63]. It is possible that the nitrogenous nutrients stored in the giant root cells could serve as a nutrient source for RKN. Detailed structural and taxa-specific genomic and transcriptomic analyses are needed in order to understand how the various partners interact with each other, during RKN parasitism.

RKN are among the most destructive pests in many crops and cause severe yield losses throughout the world [64]. Their broad host range indicates that RKN has the ability to acquire essential nutrients such as nitrogen from diverse host plants for their growth, development, and reproduction. Our finding of enriched biological nitrogen fixers in RKN-parasitized roots represents a potentially common mechanism of nutrient acquisition for RKN in plant roots. Understanding the balance of biological nitrogen fixation between plant hosts and nematodes could help develop new RKN biological control strategies, without negatively impacting host plant health and productivity. A previous study demonstrated that co-inoculation of *M. javanica* and *Rhizobium* spp. resulted in increased galls in broad beans [65]. However, studies also demonstrated that co-inoculation of RKN and rhizobia resulted in reduced galling and nodulation [58]. At present, it is not known whether different RKN species form symbiotic or mutualistic relationships with different nitrogen-fixing bacteria and whether the nitrogen fixers in RKN (i.e., in galls) are different from those involved in biological nitrogen fixation in plants such as the *Rhizobium*-legume symbiosis [22]. In addition, the potential interactions between nematode parasitism and other root diseases such as root rot in crop plants and how nitrogen-fixing bacteria influence such interactions remain unknown. Root rot is among the most common and devastating infectious diseases in crop plants and with many fungal pathogens as causative agents [66]. Knowledge obtained from investigations addressing such interactions will help us in better harnessing the potential of biological nitrogen fixation to not only reducing reliance on chemical nitrogen fertilizers, but also reducing RKN parasitism and improving overall plant health.

## Conclusions

Our work showed that multiple factors, including developmental stage, ecological niche, and RKN parasitism, contributed significantly to variations in root-associated microbiota. Specifically, community variation and assembly of root endophytic microbiota were significantly affected by RKN parasitism in a niche-specific pattern. Our results demonstrated for the first time that the taxonomic and functional associations of endophytic nitrogen-fixing bacteria with RKN parasitism in plants, and the change of nitrogen-fixing bacterial communities through the addition of nitrogen fertilizers could affect the occurrence of root-knot nematodes. Our results provided new insights into interactions among endophytic microbiota, RKN, and plants, and suggested a novel mechanism for designing biological control strategies against RKN by manipulating nitrogen levels in crop fields.

## Methods

### Sample collection from plants with and without RKN

To assess the effects of *Meloidogyne* spp. on root-associated microbiota, rhizosphere soil and root samples of healthy and parasitized plants were collected in Shunchang County of Fujian province, China (26° 38′–27° 121′ N, 117° 29′–118° 14′ E) in June 2016 (Additional Table S1). Samples of three vegetables, tomato (*Solanum lycopersicum*), lettuce (*Lactuca sativa* L. var. ramosa Hort.), and celery (*Apium graveolens* L.), were collected from a vegetable farm, monitored for RKN parasitism for at least 5 years before sample collection [67]. The field prevalence of RKN for the three crops were approximately 30–50%. Two perennial plants, Snakegourd fruit (*Trichosanthes kirilowii* Maxim.) and citrus (*Citrus reticulata* Blanco), attacked by RKN for at least 2 years (severe parasitism, with > 75% roots with galls, and swollen by > 75%), were separately collected from orchards with RKN. The collected lettuce and celery roots showed a low RKN parasitism symptom (less than one third roots with galls), and tomato root with a moderate RKN parasitism symptom (more than half of roots with galls) (Additional Table S1). At least three replicated healthy or nematode-parasitized plants were sampled for each plant species. The collected plants were used to separate rhizosphere soil and root samples for the 16S rRNA gene-based high-throughput sequencing and bacterial community analysis (Additional Table S1).

### Tomato growth and sampling

To minimize the confounding effects of plant species and their differential susceptibilities to RKN attacks on bacterial community analyses, we systematically investigated bacterial community composition around roots at the different growth and disease developmental stages using tomato as a model. Seeds of tomato cultivar Xinzhongshu No. 4, susceptible to RKN (*Meloidogyne incognita*) were surface-sterilized in 0.5% sodium hypochlorite solution for 15 min and 70% ethanol for 1 min. The sterilized seeds were rinsed extensively in sterile water five times, and then germinated in sterile plates under dark condition at 28 °C for 3 days [22, 68]. Germinated seeds were separately planted into two adjacent experimental fields at Qishan campus of Fujian Normal University in Fuzhou, Fujian province, China (26° 01′ N, 119° 12′ E) from June to August 2017. One field had no record of extensive RKN parasitism and was used to grow healthy plants. Another field was a nursery for tomato plants known to contain *M*. *incognita* for at least 3 years prior to planting, at nematode parasitism rates above 90%.

To investigate the effects of plant growth and RKN parasitism on tomato root-associated microbiota, we collected the first tomato samples at the second true leaf stage (about ten days after planting). After that, samplings were conducted every 7 days. A total of nine (for nematode-parasitized plants) or ten (for healthy tomato plants) stages were sampled. For each stage, three replicated plants were sampled for both the healthy and nematode parasitism treatments (Additional Table S3). The healthy and parasitized conditions of tomato plants were confirmed by examining the presence of RKN in small fragments of sampled roots, stained with acid fuchsin, following an established protocol [69]. The collected plant samples were used to further separate rhizosphere soil and root samples for the following 16S rRNA gene sequencing and bacterial community analysis (Additional Table S3).

### Sample separation of rhizosphere soil, roots, and/or galls

During sampling, healthy and nematode-parasitized plants were pulled carefully from soil and shaken to remove large soil particles, leaving soil that was strongly attached to the roots. Rhizosphere soil and roots were separately collected from the sampled materials as described by Edwards et al. and Kwak et al. [70, 71]. To obtain the bacterial community profiles specifically associated with RKN parasitism, galls induced by *Meloidogyne* spp. were separated from the surface-sterilized parasitized roots with a sterile scalpel [22]. Briefly, surface-sterilized parasitized roots were separated into two fractions: one contained the galls, whereas the other contained the non-swollen part of the parasitized root system. Together, for each sampled healthy plant, the rhizosphere soil and root fractions were isolated. For each nematode-parasitized plant, its rhizosphere soil and the two root fractions were obtained. In total, 75 samples (30 from rhizosphere soil, 30 from roots, and 15 from gall samples) were collected from healthy and parasitized plants, in the five different plant species (Additional Table S1). Similarly, 135 samples (57 rhizosphere soil, 57 root, and 21 gall samples) were collected from 9 or 10 growth stages, respectively, from the healthy and nematode-parasitized tomato plants (Additional Table S3).

### Genomic DNA extraction and 16S rDNA amplicon sequencing

Total genomic DNA was extracted from each rhizosphere soil or root samples using a Power Soil® DNA Isolation Kit (MoBio Laboratories, Carlsbad, CA, USA) according to the manufacturer’s instructions. To assess DNA concentration and purity, the DNA extracts were run on 1% agarose gels at 110 V for 30 min and quantified using a NanoDrop 2000 spectrophotometer (Thermo Scientific). The extracted total genomic DNA samples were stored at 20 °C until subjected to high-throughput sequencing.

Approximately 400-bp DNA fragments of the bacterial 16S rRNA gene targeting the hypervariable region V3-V4 were amplified using barcoded universal primer pair 341F (5′-CCTACGGGNGGCWGCAG-3′) and 805R (5′-GACTACHVGGGTATCTAATCC-3′) in the bacterial community analysis for the five plant species [22]. To minimize the effect of chloroplast DNA of host plant on microbiota analyses, another barcoded universal primer pair 799F (5′-AACMGGATTAGATACCCKG-3′) and 1193R (5′-ACGTCATCCCCACCTTC C-3′), spanning ~ 450 bp of the V5-V7 regions of the 16S rRNA gene, was used in the subsequent community analysis, including the analysis of tomato microbiota at different developmental stages, and of tomato amended with different nitrogen sources [35, 43, 72]. Amplified PCR products in each experiment were separately processed to purify, combined in equimolar ratios, and subjected to high-throughput sequencing on an Illumina Mi-Seq sequencing platform, and paired 250-nucleotide reads were produced at Sangon Biotech (Shanghai, China).

### Data processing, bioinformatics, and statistical analyses

The raw sequences were spliced, merged, and then filtered to remove the low-quality, chimeric, and less than 200 bp reads as described by Lei et al. and Cheng et al. [68, 73]. After chimera removal, the processed high-quality sequencing reads were then clustered into operational taxonomic units (OTUs) by USEARCH (version 10.0) based on 97% pairwise identity using the SILVA reference alignment (database release 128, updated September 2016) as a reference [74]. Taxonomic classification of the representative sequence for each OTU was done using the Ribosomal Database Project’s classifier of QIIME’s pipeline against the SILVA database, with a minimum confidence of 85% [75]. All OTUs identified as belonging to chloroplast and mitochondria were further removed from the OTU table and OTU representative sequences files. The filtered OTU tables for all samples were subsequently normalized based on library size and used for the following downstream analyses.

In this study, we first used V3-V4 primers to analyze the community composition of plant-associated bacterial taxa in the five plant species for both healthy and RKN-parasitized plants. After quality check and chimera sequence filtering, we obtained 112,908 ± 42,918 (mean ± SD) processed high-quality reads, per sample. Following chloroplast and mitochondria DNA removal, an average of 52,965 ± 34,070 effective bacterial reads per sample were obtained (Additional Table S1). The finalized reads were clustered to OTUs, yielding an average of 2864 ± 1380 OTUs per sample (range: 1089 − 5960). Subsequently, to reduce the chloroplast and mitochondrial DNA in the sequencing data, the V5-V7 primers were selected and used for the remaining 16S rRNA gene-based community analysis of plant-associated microbiota. An average of 19,240 ± 5387 effective bacterial reads per sample were obtained (Additional Table S3).

Based on the OTU numbers and the relative abundance of each OTU, the alpha diversity indexes were calculated using the QIIME pipeline [75]. For beta diversity, Bray–Curtis and UniFrac-based distances were calculated from the normalized OTU tables for each sample. PCoA (principal coordinate analysis) was generated with the Bray–Curtis distances (using R package *vegan*) [75, 76]. CAP (Canonical Analysis of Principle Coordinates) analysis was performed using the function “capscale()” from package *vegan*. Variance partitioning and significance for experimental factors were performed by running the *vegan* permutes function over the CAP model, using a maximum of 999 permutations [43, 77]. Furthermore, variance partitioning analysis (VPA) was used to quantify the relative importance of the explanatory variables: compartments (niche), nematode parasitism (nemato), and developmental stages (days) to bacterial community composition [78]. The Pearson correlation coefficients were calculated by a “cor()” function and visualized by using the R package *corrplot* [72].

To infer the community assembly processes in the healthy and nematode-parasitized tomato microbiota, the mean nearest taxon distance metric was calculated using the R package picante with the null modeling approach, to calculate the β-nearest taxon index (βNTI) [34, 79–81]. The value of |βNTI|> 2 is commonly used to indicate that observed turnover between a pair of communities is governed primarily by deterministic processes, whereas a |βNTI| value < 2 commonly indicates that the pairwise community differences are likely the result of stochastic processes [79, 81].

To discriminate the patterns of change for taxa across healthy and parasitized plants over time, the relative abundances of bacterial taxa at the order level against developmental time was regressed in the field using the R package *randomForest* with default parameters [35, 72]. Here, the Random Forest machine-learning method was first used to correlate root microbiota composition with RKN parasitism at the order level. Ten-fold cross-validation was performed with three repetitions to evaluate the importance of indicator bacterial orders to differentiate healthy and parasitized samples. The classification model was generated and the number of marker taxa was identified. To statistically analyze the abundances of individual OTUs, the means and standard deviations were calculated in each taxonomic count dataset. Wilcoxon rank sum test was utilized to identify taxa significantly different among different treatments or groups of treatments, at different taxonomic (including OUT) levels. The probability values were adjusted for multiple tests using a false discovery rate (FDR) at 0.05. Statistical analysis was performed using analysis of variance (ANOVA) with *p* values and post-test with TukeyHSD to determine whether the diversity indexes were statistically significant among the different treatments. Additionally, permutational MANOVA was carried out to using *vegan*’s function “adonis()” to measure effect size and significances on β-diversity. To infer functional differentiation of bacterial taxa among samples, potential functions among members of the bacterial community were annotated based on FAPROTAX, and functions with significant differences (*p* < 0.05) in relative abundance were identified by STAMP [82, 83]. The OTUs related to the enriched pathways were then determined and compared to find the functionally attributes of differential bacterial taxa in related to the occurrence of nematodes in plants [35, 36].

### Microbiome enrichment, protein extraction, and metaproteomic analysis

For metaproteomic analysis, tomato plants were sown and grown in the experimental fields from May 2018 according to the described procedures above. At the second true leaf stage (about ten days), tomato plants were separately transplanted and grown in experimental fields with and without prior RKN parasitism. The first tomato samples were collected at the 14th day after transplanting, and then samplings were conducted every 2 weeks, three more times. Finally, a total of four growth stages for healthy or nematode-parasitized tomato plants were sampled (Additional Table S11). For each stage, 30–50 healthy or nematode-parasitized tomato plants were collected, and then the healthy and parasitized roots (non-gall and gall fractions) were separated as described above. The collected samples for each treatment were separately combined and used for the enrichment of endophytic microbiota as described by Tian et al. [22]. Briefly, the surface-sterilized roots or galls were soaked in a pectinase solution, and gently mashed with a pestle to crush. The homogenized solution was filtered through a series of polycarbonate filters (Millipore, 100-, 25-, 10-, and 7-μm) under vacuum and then the filtrate was centrifuged to obtain endophytic bacterial sediment [22]. Finally, 12 endophytic bacterial sediment samples (from 4 healthy roots, 4 parasitized roots, and 4 gall samples) were obtained (Additional Table S11). Proteins were extracted from the enriched endophytic microbiota according to a modified phenol method [84]. The protein pellet obtained was washed with cold methanol for three times, then with acetone once. The protein precipitate was re-dissolved in 8 M urea and 100 mM borane-triethylamine (TEAB, pH 8.0), and then the concentration was quantified by the BCA protein assay method [84].

The extracted total proteins from the enriched endophytic microbiota were trypsin digested into peptides and subsequently analyzed by liquid chromatography tandem mass spectrometry (LC–MS/MS) in Q ExactiveTM Plus (Thermo) coupled online to the UPLC [84]. The resulting MS/MS data were analyzed using Maxquant search engine (v.1.5.2.8) against the in-house database for protein identification with reverse decoy database. FDR was adjusted to < 1%, and minimum score for modified peptides was set > 40. The parameters were set according to methods described [84]. For proteomic analysis, bacteria-derived proteins were specially separated using the BLAST program against the bacteria-derived sub-database from the non-redundant protein database (NR NCBI). Functional annotations of proteins were conducted using InterProScan (v.5.14) against UniProt-GOA database, based on a protein sequence alignment method. The GO annotation and KEGG database were used to annotate and group these identified proteins.

### Quantitative analysis and fluorescence localization for nitrogenase nifH gene in Meloidogyne-parasitized tomato roots

To investigate the copy number of nitrogenase gene in root endophytic microbiota during RKN parasitism, we performed quantitative PCR (qPCR) for the nitrogenase gene *nifH*. Considering the diverse nitrogenase genes, the genomic DNA previously extracted from rhizosphere soil and root tissue samples were first used to screen appropriate primer pairs for nitrogenase gene *nifH* amplification according to the published literatures [38, 85–88]. Finally, primer pair nifHF (5′-AAAGGYGGWATCGGYAARTCCACCAC-3′) and nifHR (5′-TTGTTSGCSGCRTACATSGCCATCAT-3′) were identified to successfully amplify *nifH* across different stages of healthy and parasitized tomato root samples [88]. Specifically, in this analysis, the extracted DNA was diluted using the sample with the lowest DNA concentration as a standard. Quantitative PCR for *nifH* gene and for the V5-V7 fragment of bacterial 16S rRNA gene (use as reference) were performed using QuantStudio 3 Real-Time PCR System (Applied Biosystems), with Power SYBR green PCR master mix (Applied Biosystems). The PCR cycling program included a 30-s pre-incubation at 95 °C, 40 cycles consisting of 95 °C for 5 s and 60 °C for 30 s for amplification. Additional reaction was performed to yield a melting curve that was used to confirm the amplification specificity. Threshold cycle (Ct) values were determined in triplicate for each sample. qPCR efficiency and sample Ct values were used to calculate the fold change to estimate the relative copy number of *nifH* gene in different samples, using the 2^−ΔΔCt^ method [89, 90].

To observe nitrogen-fixing bacteria in root tissues and to demonstrate their association with nematodes, the *nifH* gene was localized by fluorescence in situ hybridization (FISH) using a fluorescence-labeled *nifH* gene fragment as probe in nematode-parasitized tomato roots [91–93]. For this assay, healthy and nematode-parasitized tomato plants were first harvested and roots with obvious gall symptoms were separated as described above. The collected roots were treated, formaldehyde fixed, paraffin wax embedded, and then sliced with a minor modification [89–91]. In detail, root tissue slices were stored at 4 °C for the subsequent probe hybridization. For FISH, digoxigenin labeled probe targeting a fragment of the *nifH* gene was prepared with primers nifHF/nifHR using the Nick Translation Kit according to the instruction of the manufacturer (Roche, Mannheim, Germany). Root slices were carefully fixed on grass slides to avoid tissue damage, and treated through the following steps, including paraffin removal, rehydration, and lysozyme permeabilization, as described [89–91]. Samples were then hybridized with the oligonucleotide probe against *nifH* gene, and then incubated with fluorescently labeled antibody. Subsequently, samples were washed and observed in a Confocal Laser Scanning Microscopy (Leica Microsystems Ltd TCSSP8, Wetzlar, Germany).

### RKN biocontrol using biofertilizer and various nitrogen sources

To reveal the association between RKN parasitism and the variation in endophytic nitrogen-fixing bacteria, seedlings of tomato cultivar cv Xinzhongshu No.4 were planted in *Meloidogyne* sp.-parasitized soils by supplying different nitrogen sources, in pot experiments carried out from June to August 2020. The soil used was collected from a nursery field with a 3-year nematode parasitism history. In total, 11 different inorganic or organic nitrogen compounds and two biofertilizers were selected for testing (Additional Table S9). Nitrogen sources were separately applied to each plot at 300 mg N/Kg soil after tomato seeding (keeping 5 tomato plants per pot out of 8–10 seeds sowed). The two biofertilizers were fresh chicken manure (fermented) and commercial chicken manure-based biofertilizer. Each nitrogen amendment treatment was performed with three replicates. Pot-planted tomato plants in soil without nematode parasitism history were used as positive control, using tomato plants in soil with nematode parasitism history but no nitrogen supplementation as negative control. At 55 days after seeding, tomato plants were harvested for the evaluation of RKN parasitism, quantifying the attack severity using the number of galls per plant [22, 49]. Subsequently, root and/or gall samples were separately collected from healthy or nematode-parasitized tomato plants, as described above. Together, 57 samples (45 root, and 12 gall samples) were collected from healthy and nematode-parasitized tomato plants, including healthy control, parasitized control, and plants treated with 13 different nitrogen sources (Additional Table S9). Furthermore, community analysis for the effect of nitrogen supplement on root endophytic microbiota was performed, following the procedure described above.

## Supplementary Information

**Additional file 1: Table S1.** Experimental design and basic sequencing statistics for samples collected from five plant species. **Table S3.** Experimental design and basic sequencing statistics for samples collected from tomato plants at different growth stages. **Table S9.** Experimental design and basic traits for samples collected from tomato plants amended with different nitrogen sources. **Table S11.** Experimental design and basic sequencing statistics from metaproteomic analyses. **Figure S1.** Order-level community comparison and OTU-based hierarchical clustering analysis of healthy and nematode parasitized samples in five plant species. **Figure S2.** Unconstrained PCoA (for principal coordinates PCo1 and PCo2) with Bray–Curtis distance showing the root-associated bacteria of healthy and parasitized samples, for each plant species. **Figure S3.** The composition and relative abundance of major bacterial orders of the tomato root-associated microbiota at different developmental stages. **Figure S4.** Pairwise analysis for the variation of root-associated microbiota over time at different growth and RKN parasitism stages in tomato. **Figure S5.** The shared OTUs number and taxonomic composition of the depleted and enriched OUTs in the parasitized root samples (IRH and IRK) comparing with healthy roots (HRC).

**Additional file 2: Table S2.** Summary of the differential bacterial communities between the rhizosphere (soil) and endophytic (root) microbiota in five plant species at the Phylum and Order levels.

**Additional file 3: Table S4.** Summary of the differential bacterial communities at the Order level between different treatments (soil and root, healthy and parasitized) at the different growth stages of tomato plants.

**Additional file 4: Table S5.** Summary of differential OTUs between the healthy and parasitized root endophytic microbiota in tomato plants.

**Additional file 5: Table S6.** Summary of differential functional pathways between healthy and parasitized root samples in tomato plants.

**Additional file 6: Table S7.** Identification of OTUs related to biological nitrogen-fixation and plant pathogens in tomato root-associated microbiota.

**Additional file 7: Table S8.** Specific proteins identified in parasitized root samples of tomatoes from metaproteomic analysis.

**Additional file 8: Table S10.** Summary of differential OTUs between healthy and parasitized root samples collected from tomato plants amended with different nitrogen sources.

## Data Availability

The sequences generated in this study have been deposited in GenBank and the NCBI Short Read Archive under BioProject number PRJNA825697, which comprises 267 SRA files for Illumina sequencing reads of the 16S rRNA gene (accession number: SRR18739670-SRR18739936).
