# Supplementary material for: Microbiota and functional analyses of nitrogen-fixing bacteria in root-knot nematode parasitism of plants
Source: Microbiome. 2023 Mar 10;11:48. doi: 10.1186/s40168-023-01484-3 (PMC9999639; doi:10.1186/s40168-023-01484-3)
Supplement: Supplementary file 2 — Additional file 1: Table S1. Experimental design and basic sequencing statistics for samples collected from five plant species. Table S3. Experimental design and basic sequencing statistics for samples collected from tomato plants at different growth stages. Table S9. Experimental design and basic traits for samples collected from tomato plants amended with different nitrogen sources. Table S11. Experimental design and basic sequencing statistics from metaproteomic analyses. Figure S1. Order-level community comparison and OTU-based hierarchical clustering analysis of healthy and nematode parasitized samples in five plant species. Figure S2. Unconstrained PCoA (for principal coordinates PCo1 and PCo2) with Bray–Curtis distance showing the root-associated bacteria of healthy and parasitized samples, for each plant species. Figure S3. The composition and relative abundance of major bacterial orders of the tomato root-associated microbiota at different developmental stages. Figure S4. Pairwise analysis for the variation of root-associated microbiota over time at different growth and RKN parasitism stages in tomato. Figure S5. The shared OTUs number and taxonomic composition of the depleted and enriched OUTs in the parasitized root samples (IRH and IRK) comparing with healthy roots (HRC). [file 40168_2023_1484_MOESM1_ESM.doc]

**Supporting Text: Detailed Results**

**Community variation in root-associated microbiota along the growth stages of tomato**

As described in the main text, bacterial communities of root-associated microbiota notably varied along the growth stages of tomato. In detail, the community analysis of tomato root-associated bacteria clearly showed that communities varied dramatically across different developmental stages, in each treatment (Fig. 2A). Specifically, taxa in bacterial Orders Rhizobiales, Betaproteobacteriales, Streptomycetales, Pseudomonadales, Micrococcales, Xanthomonadales and Corynebacteriales rapidly colonized the root tissues in the early stage (about 17-21 days) (Fig. 2A; Additional Fig. S3). Several bacterial taxa were correlated to specific growth stages of tomato plants, such as Enterobacteriales, which were highly enriched in R4-R5 (38-45 days), but dramatically decreased in later stages. Compared to healthy roots, in nematode-parasitized roots (IRH and IRK), taxa in Rhizobiales were highly enriched from the first sampling onward (Additional Fig. S3). Finally, OTUs in Betaproteobacteriales, Rhizobiales, Xanthomonadales and Pseudomonadales (Proteobacteria), and Corynebacteriales, Pseudonocardiales, and Streptomycetales (Actinobacteria) constituted most of the stable root endophytic bacterial taxa, consistent with a previous report on tomato plants (Fig. 2A; Additional Fig. S3) [22]. In contrast, bacterial community changes across different plant developmental stages were less pronounced in the rhizosphere soil. Most of the identified bacterial taxa in the last collected soil samples were also found in the samples taken at the initial time point (Fig. 2A; Additional Fig. S3A). PCoA results also showed that the microbial community variation in root-associated taxa was differentiated along tomato growth stages for some samples, but not for others. Specifically, root samples from healthy tomato plants (HRC) showed a notable temporal pattern along the first PCoA axis while those from the rhizosphere soil (HRS and IRS) and from parasitized tomato roots (IRH and IRK) showed no consistent temporal distributions (Fig. 2B).

To further reveal the microbial community variation in root-associated microbiota along the growth stages of tomato, community similarities were evaluated using correlation and Bray-Curtis distance analyses (Additional Fig. S4A-B). The pairwise correlation analysis between pairs of samples revealed that the community compositions of root-associated microbiota showed greater variations in earlier developmental stages, but that they gradually stabilized and became increasingly similar to each other over time (Additional Fig. S4B). Analyses of pairwise Bray-Curtis distances further revealed that the community variation of root-associated bacteria in each treatment was significantly related to the sampling time distance (Additional Fig. S4A; *p* < 0.001).

**Additional file: Tables and Figures:**

Table S1. Experimental design and basic sequencing statistics for samples collected from five plant species.

Table S2. Summary of the differential bacterial communities between the rhizosphere (soil) and endophytic (root) microbiota in five plant species at the Phylum and Order levels.

Table S3. Experimental design and basic sequencing statistics for samples collected from tomato plants at different growth stages.

Table S4. Summary of the differential bacterial communities at the Order level between different treatments (soil and root, healthy and parasitized) at the different growth stages of tomato plants.

Table S5. Summary of differential OTUs between the healthy and parasitized root endophytic microbiota in tomato plants.

Table S6. Summary of differential functional pathways between healthy and parasitized root samples in tomato plants.

Table S7. Identification of OTUs related to biological nitrogen-fixation and plant pathogens in tomato root-associated microbiota.

Table S8. Specific proteins identified in parasitized root samples of tomatoes from metaproteomic analysis.

Table S9. Experimental design and basic traits for samples collected from tomato plants amended with different nitrogen sources.

Table S10. Summary of differential OTUs between healthy and parasitized root samples collected from tomato plants amended with different nitrogen sources.

Table S11. Experimental design and basic sequencing statistics from metaproteomic analyses**.**

**Additional files**

**Table S1.** **Experimental design and basic sequencing statistics for samples collected from five plant species.**

| **Sample ID** | **Plant species** | **Niches** | **Treatments** | **Life_cycle** | **Galls** | **Processed#** | **Effective#** |
| --- | --- | --- | --- | --- | --- | --- | --- |
| JFG | Tomato | Root | Healthy root (HR) | annual | - | 117610 | 26338 |
| CFG | Tomato | Root | Parasitized root (PR) | annual | moderate | 111098 | 28463 |
| CFL | Tomato | Root | Galls (GR) | annual | moderate | 152045 | 26663 |
| JFT | Tomato | Soil | Healthy soil (HS) | annual | - | 92884 | 92532 |
| CFT | Tomato | Soil | Soil with RKN (PS) | annual | moderate | 92296 | 91361 |
| JGG | Snakegourd fruit | Root | Healthy root (HR) | perennial | - | 176582 | 15342 |
| CGG | Snakegourd fruit | Root | Parasitized root (PR) | perennial | severe | 119546 | 29876 |
| CGL | Snakegourd fruit | Root | Galls (GR) | perennial | severe | 52505 | 21639 |
| JGT | Snakegourd fruit | Soil | Healthy soil (HS) | perennial | - | 107184 | 106758 |
| CGT | Snakegourd fruit | Soil | Soil with RKN (PS) | perennial | severe | 90618 | 90421 |
| JJG | Citrus | Root | Healthy root (HR) | perennial | - | 122509 | 20681 |
| CJG | Citrus | Root | Parasitized root (PR) | perennial | severe | 80462 | 38426 |
| CJL | Citrus | Root | Galls (GR) | perennial | severe | 113295 | 29369 |
| JJT | Citrus | Soil | Healthy soil (HS) | perennial | - | 100488 | 100436 |
| CJT | Citrus | Soil | Soil with RKN (PS) | perennial | severe | 101576 | 101520 |
| JSG | Lettuce | Root | Healthy root (HR) | annual | - | 123266 | 34005 |
| CSG | Lettuce | Root | Parasitized root (PR) | annual | slight | 90616 | 40966 |
| CSL | Lettuce | Root | Galls (GR) | annual | slight | 95427 | 38478 |
| JST | Lettuce | Soil | Healthy soil (HS) | annual | - | 74631 | 74295 |
| CST | Lettuce | Soil | Soil with RKN (PS) | annual | slight | 86417 | 86248 |
| JQG | Celery | Root | Healthy root (HR) | annual | - | 187007 | 28962 |
| CQG | Celery | Root | Parasitized root (PR) | annual | slight | 183435 | 16054 |
| CQL | Celery | Root | Galls (GR) | annual | slight | 177519 | 12581 |
| JQT | Celery | Soil | Healthy soil (HS) | annual | - | 84042 | 83396 |
| CQT | Celery | Soil | Soil with RKN (PS) | annual | slight | 89644 | 89326 |
|  |  |  |  |  |  |  |  |

Notes: The experiment was performed with three replicates for each treatment.

# Processed: mean reads number for three replicates of each treatment after qualified and chimera sequences filtering. Effective: mean reads number for three replicates of each treatment after chloroplast sequences filtering.

**Table S3.** **Experimental design and basic sequencing statistics for samples collected from tomato plants at different growth stages.**

| **Sample ID** | **Days*** | **Niches** | **Treatments** | **Processed#** | **Effective#** |
| --- | --- | --- | --- | --- | --- |
| HRC1 | 10 | Root | Healthy root (HRC) | 22667 | 20760 |
| HRC2 | 17 | Root | Healthy root (HRC) | 21333 | 19388 |
| HRC3 | 24 | Root | Healthy root (HRC) | 112809 | 8068 |
| HRC4 | 31 | Root | Healthy root (HRC) | 45997 | 8443 |
| HRC5 | 38 | Root | Healthy root (HRC) | 47131 | 19771 |
| HRC6 | 45 | Root | Healthy root (HRC) | 62010 | 10834 |
| HRC7 | 52 | Root | Healthy root (HRC) | 57370 | 10935 |
| HRC8 | 59 | Root | Healthy root (HRC) | 29215 | 16761 |
| HRC9 | 66 | Root | Healthy root (HRC) | 37155 | 21855 |
| HRC10 | 73 | Root | Healthy root (HRC) | 36190 | 22094 |
| HRS1 | 10 | Soil | Healthy soil (HRS) | 23524 | 23479 |
| HRS2 | 17 | Soil | Healthy soil (HRS) | 25202 | 25165 |
| HRS3 | 24 | Soil | Healthy soil (HRS) | 27470 | 27440 |
| HRS4 | 31 | Soil | Healthy soil (HRS) | 25812 | 25650 |
| HRS5 | 38 | Soil | Healthy soil (HRS) | 29205 | 28925 |
| HRS6 | 45 | Soil | Healthy soil (HRS) | 25556 | 25379 |
| HRS7 | 52 | Soil | Healthy soil (HRS) | 22896 | 22590 |
| HRS8 | 59 | Soil | Healthy soil (HRS) | 23361 | 23037 |
| HRS9 | 66 | Soil | Healthy soil (HRS) | 25083 | 24833 |
| HRS10 | 73 | Soil | Healthy soil (HRS) | 18787 | 18653 |
| IRH1 | 10 | Root | Parasitized root (IRH) | 22360 | 21732 |
| IRH2 | 17 | Root | Parasitized root (IRH) | 21876 | 21599 |
| IRH3 | 24 | Root | Parasitized root (IRH) | 24289 | 24261 |
| IRH4 | 31 | Root | Parasitized root (IRH) | 22237 | 22230 |
| IRH5 | 38 | Root | Parasitized root (IRH) | 19492 | 19407 |
| IRH6 | 45 | Root | Parasitized root (IRH) | 20131 | 20114 |
| IRH7 | 52 | Root | Parasitized root (IRH) | 19165 | 19159 |
| IRH8 | 59 | Root | Parasitized root (IRH) | 19850 | 19639 |
| IRH9 | 66 | Root | Parasitized root (IRH) | 25043 | 24973 |
| IRK3 | 24 | Root | Parasitized root gall (IRK) | 24857 | 24829 |
| IRK4 | 31 | Root | Parasitized root gall (IRK) | 20633 | 19882 |
| IRK5 | 38 | Root | Parasitized root gall (IRK) | 17773 | 16835 |
| IRK6 | 45 | Root | Parasitized root gall (IRK) | 19564 | 16863 |
| IRK7 | 52 | Root | Parasitized root gall (IRK) | 22977 | 22607 |
| IRK8 | 59 | Root | Parasitized root gall (IRK) | 21778 | 21776 |
| IRK9 | 66 | Root | Parasitized root gall (IRK) | 20852 | 20806 |
| IRS1 | 10 | Soil | Soil with RKN (IRS) | 11398 | 11357 |
| IRS2 | 17 | Soil | Soil with RKN (IRS) | 11706 | 11691 |
| IRS3 | 24 | Soil | Soil with RKN (IRS) | 12866 | 12797 |
| IRS4 | 31 | Soil | Soil with RKN (IRS) | 10996 | 10957 |
| IRS5 | 38 | Soil | Soil with RKN (IRS) | 11723 | 11678 |
| IRS6 | 45 | Soil | Soil with RKN (IRS) | 10930 | 10883 |
| IRS7 | 52 | Soil | Soil with RKN (IRS) | 15481 | 15436 |
| IRS8 | 59 | Soil | Soil with RKN (IRS) | 18346 | 18310 |
| IRS9 | 66 | Soil | Soil with RKN (IRS) | 22015 | 21944 |
|  |  |  |  |  |  |

Notes: The experiment was performed with three replicates for each treatment.

* Days start from the seeding of tomato.

# Processed: mean reads number for three replicates of each treatment after the qualified and chimera sequences filtering. Effective: mean reads number for three replicates of each treatment after chloroplast sequences filtering.

**Table S9.** **Experimental design and basic traits for samples collected from tomato plants amended with different nitrogen sources.**

| **Sample ID** | **Nitrogen Treatment** | **Compartments** | **Groups** | **Gall numbers** | **Soil nitrogen content** (mg/g) |
| --- | --- | --- | --- | --- | --- |
| AR | Control | Root | Healthy | 0 (n=8) | 0.60±0.04 |
| BR | Control | Root (no gall) | Parasitized | 8±1 (n=8) | 0.53±0.05 |
| BK | Control | Gall | Parasitized |
| CR | NH_4_Cl | Root (no gall) | Parasitized | 37±12.12 (n=3) | 0.69±0.09 |
| CK | NH_4_Cl | Gall | Parasitized |
| DR | (NH_4_)_2_CO_3_ | Root (no gall) | Parasitized | 22.5±14.18* (n=4) | 0.86±0.15* |
| DK | (NH_4_)_2_CO_3_ | Gall | Parasitized |
| ER | NH_3_·H_2_O | Root (no gall) | Parasitized | 25±6.22** (n=4) | 0.59±0.06 |
| EK | NH_3_·H_2_O | Gall | Parasitized |
| FR | (NH_4_)_2_SO_4_ | Root | Healthy | 0** (n=6) | 0.90±0.12* |
| GR | (NH_4_)_2_HPO_4_ | Root | Healthy | 0** (n=8) | 0.82±0.10* |
| HR | NH_4_NO_3_ | Root | Healthy | 0** (n=8) | 1.08±0.08** |
| IR | KNO_3_ | Root | Healthy | 0** (n=7) | 1.40±0.24** |
| JR | NaNO_3_ | Root | Healthy | 0** (n=5) | 0.54±0.03 |
| KR | Ca(NO_3_)_2_ | Root | Healthy | 0** (n=8) | 0.64±0.10 |
| LR | CO(NH_2_)_2_ | Root | Healthy | 0** (n=8) | 0.99±0.19* |
| MR | CaCN_2_ | Root | Healthy | 0** (n=8) | 0.76±0.14 |
| NR | Fresh fecal | Root | Healthy | 0** (n=8) | 1.96±0.09** |
| OR | Biofertilizer | Root | Healthy | 0** (n=8) | 1.00±0.03** |

Notes: Bacterial 16S rDNA-based community and soil nitrogen content analysis were performed with three replicates for each treatment (n=3).
Significance: *: *p* < 0.05; **: *p*< 0.01.

**Table S11.** **Experimental design and basic sequencing statistics from metaproteomic analyses.**

| **Sample ID** | **Days*** | **Treatments** | **Compartments** | **Identified**# | **Quantifiable**# |
| --- | --- | --- | --- | --- | --- |
| HRE-1 | 14 | Healthy | Root | 346 | 208 |
| NKHRE-1 | 14 | Parasitized | Root (non-gall) | 150 | 114 |
| NKRE-1 | 14 | Parasitized | Root gall | 150 | 116 |
| HRE-2 | 28 | Healthy | Root | 346 | 208 |
| NKHRE-2 | 28 | Parasitized | Root (non-gall) | 150 | 116 |
| NKRE-2 | 28 | Parasitized | Root gall | 150 | 116 |
| HRE-3 | 42 | Healthy | Root | 346 | 208 |
| NKHRE-3 | 42 | Parasitized | Root (non-gall) | 150 | 115 |
| NKRE-3 | 42 | Parasitized | Root gall | 150 | 116 |
| HRE-4 | 56 | Healthy | Root | 346 | 208 |
| NKHRE-4 | 56 | Parasitized | Root (non-gall) | 150 | 116 |
| NKRE-4 | 56 | Parasitized | Root gall | 150 | 116 |
|  |  |  |  |  |  |

* Days start from the transplantation of tomato plants.

# Identified: number of identified bacterial proteins for each sample from metaproteomic analyses; Quantifiable: number of quantifiable bacterial proteins for each sample from metaproteomic analyses.

**Additional Figures:**

**Figure S1 Order-level community comparison and OTU-based** **hierarchical clustering analysis of healthy and nematode parasitized samples in five plant species.** (A) The composition and relative abundance of major bacterial taxa in five plant species at order level. (B) OTU-based hierarchical clustering analysis with Bray-Curtis distance showing the rhizosphere and endophytic microbiota of the samples with/without RKN, from five different plant species. For descriptions of sample groups, see Figure 1.


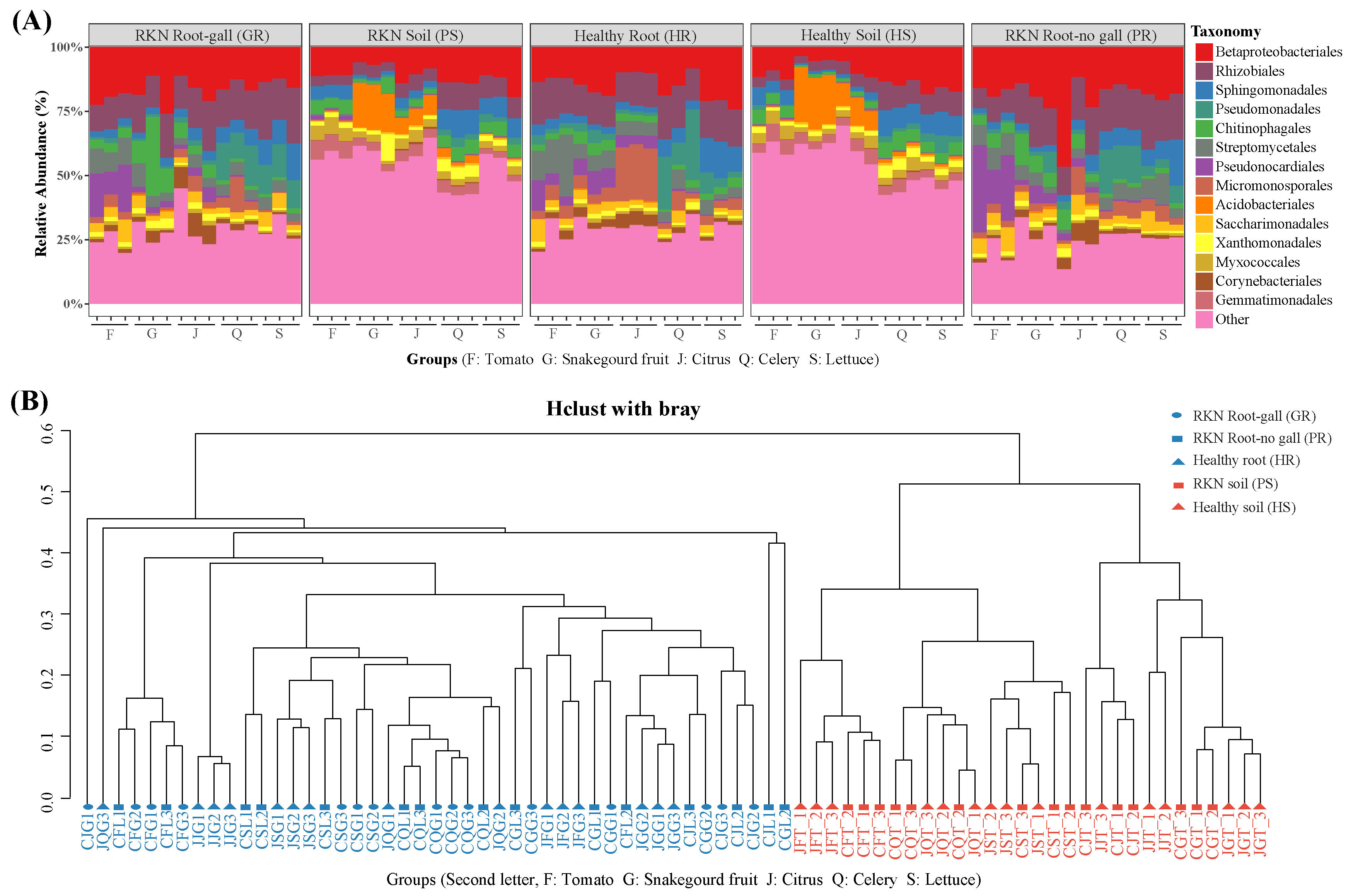


**Figure S2 Unconstrained PCoA (for principal coordinates PCo1 and PCo2) with Bray–Curtis distance showing the root-associated bacteria of healthy and parasitized samples, for each plant species**. (A) Tomato; (B) Lettuce; (C) Snakegourd fruit; (D) Citrus; (E) Celery. For description of sample groups, see Figure 1.


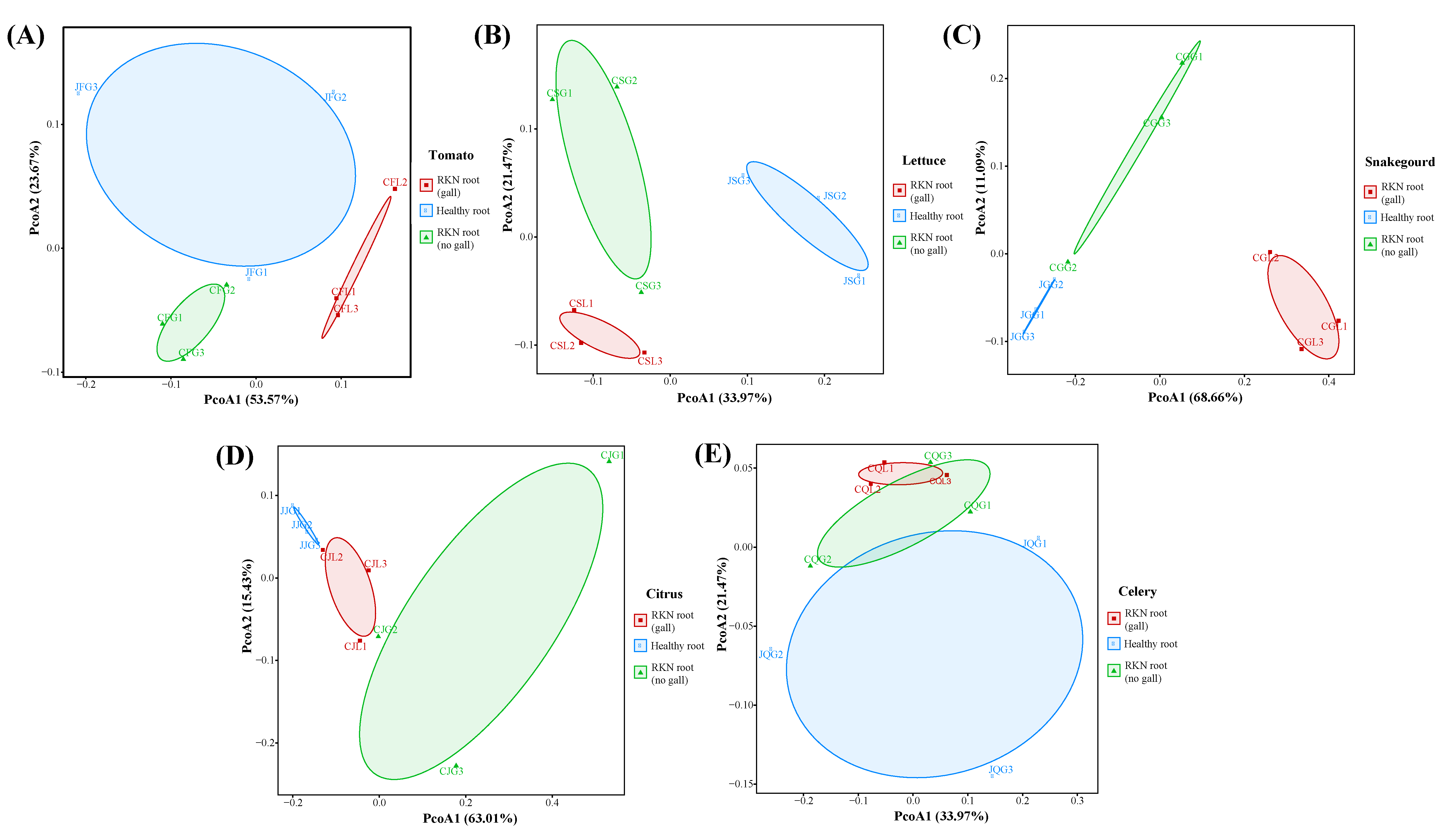


**Figure S3. The composition and relative abundance of major bacterial orders of the tomato root-associated microbiota at different developmental stages.** For description of sample groups, see Figure 2.

**
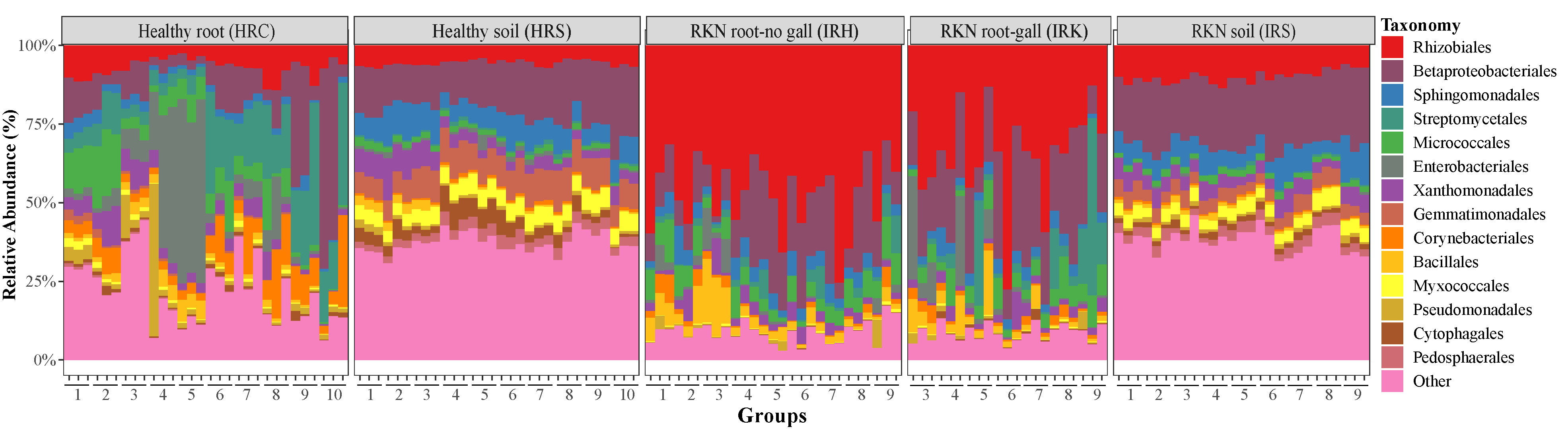
**

**Figure S4**. **Pairwise analysis for the variation of root-associated microbiota over time at different growth and RKN parasitism stages in tomato. (**A) Changes in Bray-Curtis distance of root-associated microbiota with increasing time in tomato (HRC: *R^2^* = 0.35, *p* < 0.001; HRS: *R^2^* = 0.52, *p* < 0.001; IRH: *R^2^* = 0.19, *p* < 0.001; IRK: *R^2^* = 0.11, *p* < 0.001; IRS: *R^2^* = 0.27, *p* < 0.001). (B) Pairwise correlation analysis of root-associated sequence/taxa representation between each pair of replicated tomato sample plots. (C) Pairwise analysis for the variation of Shannon (HRC: *R^2^* = 0.61, *p* < 0.001; HRS: *R^2^* = 0.13, *p* = 0.312; IRH: *R^2^* = 0.22, *p* = 0.123; IRK: *R^2^* = 0.05, *p* = 0.835; IRS: *R^2^* = 0.39, *p* = 0.008), Chao1 (HRC: *R^2^* = 0.72, *p* < 0.001; HRS: *R^2^* = 0.24, *p* = 0.063; IRH: *R^2^* = 0.49, *p* = 0.001; IRK: *R^2^* = 0.42, *p* = 0.024; IRS: *R^2^* = 0.16, *p* = 0.244) and observed species (HRC: *R^2^* = 0.79, *p* < 0.001; HRS: *R^2^* = 0.22, *p* = 0.091; IRH: *R^2^* = 0.24, *p* = 0.087; IRK: *R^2^* = 0.15, *p* = 0.414; IRS: *R^2^* = 0.37, *p* = 0.012) indexes of root-associated microbiota along the time gradient applied. For description of sample groups, see Figure 2.


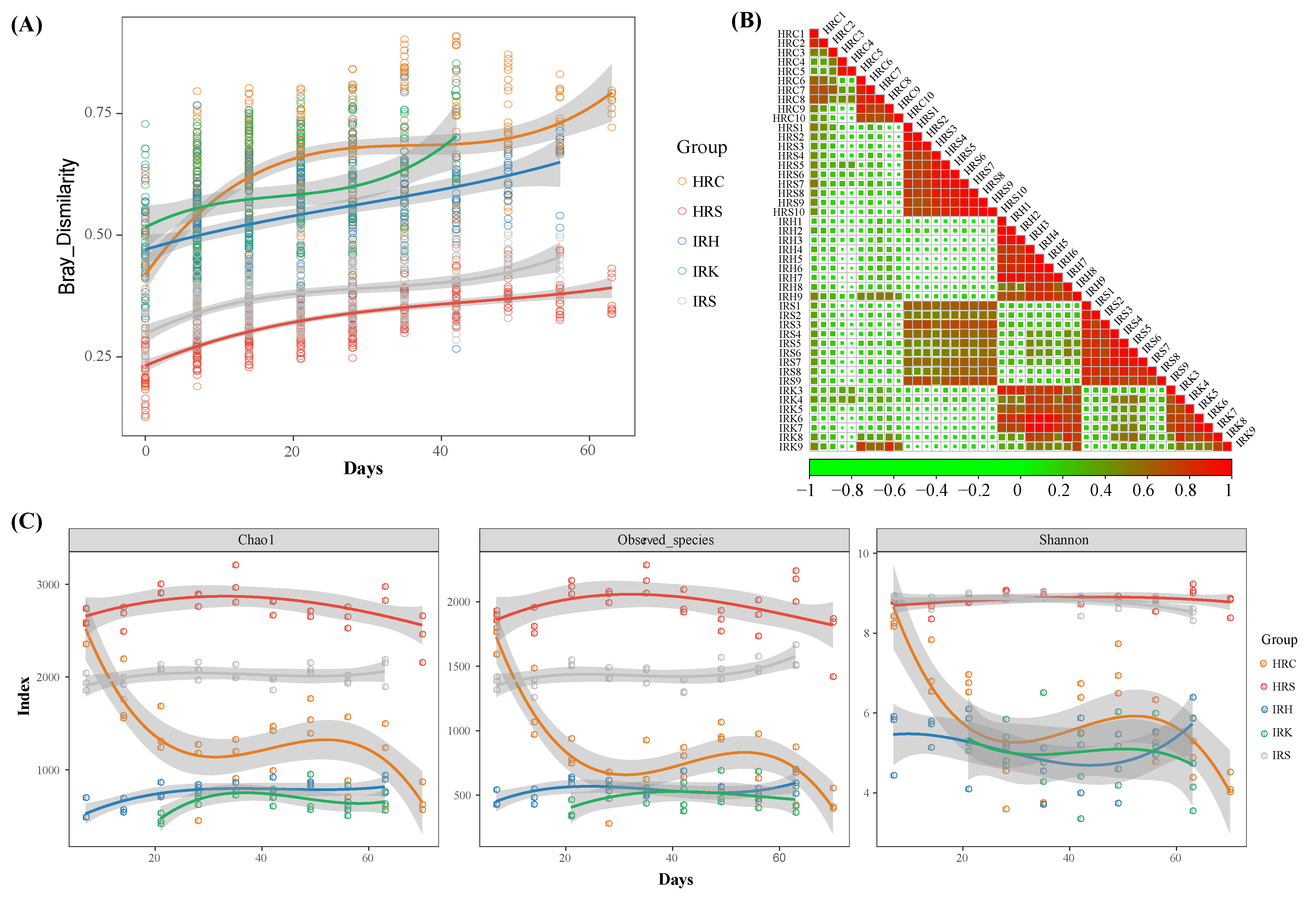


**Figure S5. The shared OTUs number and taxonomic composition of the depleted and enriched OUTs in the parasitized root samples (IRH and IRK) comparing with healthy roots (HRC)**. (A-B) Venn diagram showing the overlapping of the depleted OTUs and their taxonomic composition in IRH-HRC and IRK-HRC. (C-D) Venn diagram showing the overlapping of the enriched OTUs and their taxonomic composition in IRH-HRC and IRK-HRC.

**
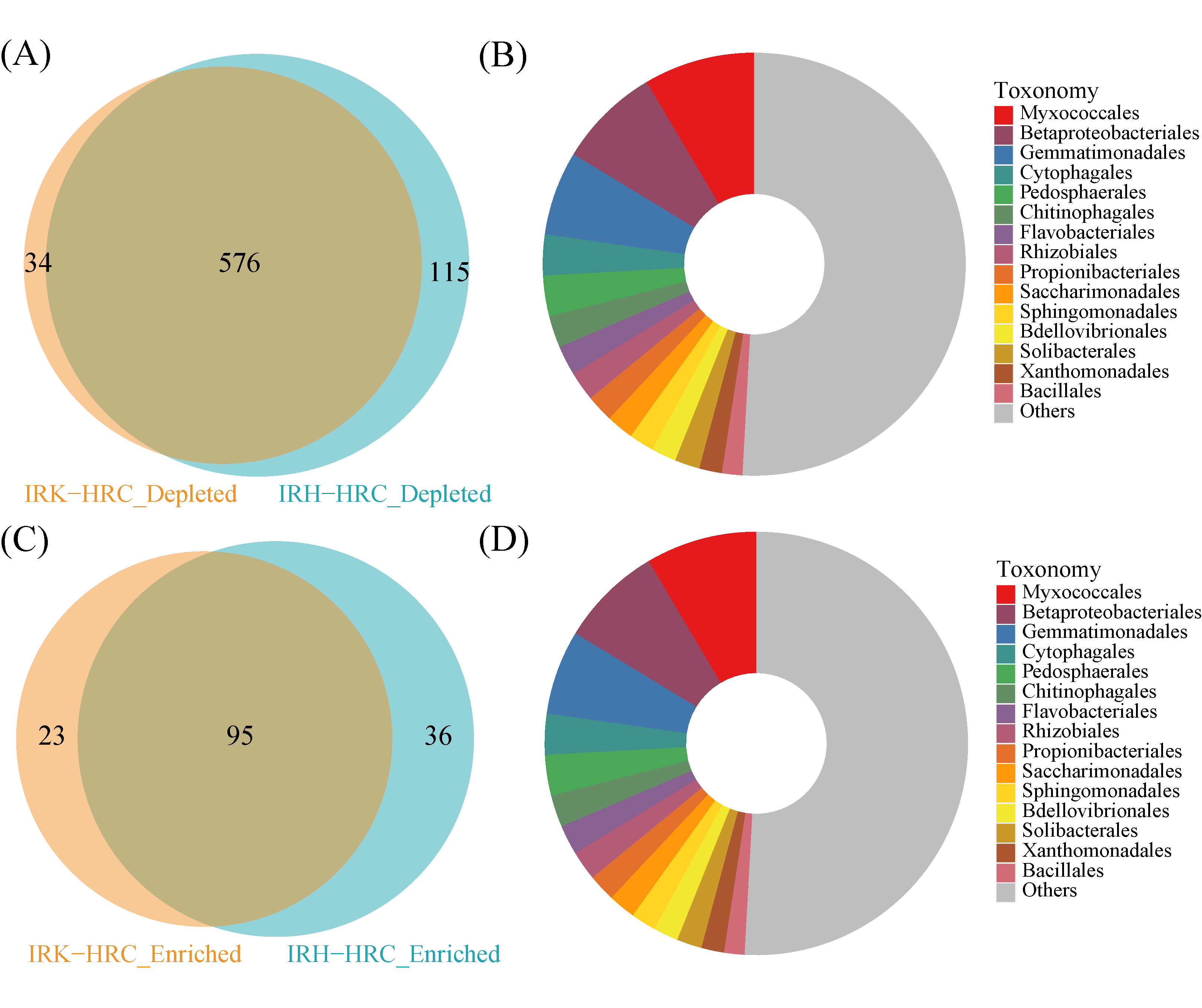
**
